# Induced variations of ethyl methane sulfonate mutagenized cowpea (*Vigna unguiculata* L. walp) plants

**DOI:** 10.3389/fpls.2022.952247

**Published:** 2022-08-05

**Authors:** Muhammed Opoku Gyamfi, John Saviour Yaw Eleblu, Lawrencia Gyamfi Sarfoa, Isaac Kojo Asante, Frank Opoku-Agyemang, Eric Yirenkyi Danquah

**Affiliations:** ^1^West Africa Centre for Crop Improvement, College of Basic and Applied Sciences, University of Ghana, Accra, Ghana; ^2^Department of Crop Science, School of Agriculture, University of Ghana, Accra, Ghana; ^3^Biotechnology Centre, College of Basic and Applied Sciences, University of Ghana, Accra, Ghana; ^4^Department of Plant and Environmental Biology, University of Ghana, Accra, Ghana

**Keywords:** mutation, variability, EMS, M1 generation, M2 generation

## Abstract

Unique variants are desired in the development of genetically improved crops to meet farmer and market needs hence ethyl methane sulfonate (EMS) was used to induce genetic variability in cowpea (*Vigna unguiculata* cv. *Asontem*). The main objective of this research was to characterize induced variations in EMS chemically mutagenized population of cowpea (*Vigna unguiculata* L. Walp Var. Asontem) in the M_1_ and M_2_ generations. The optimum concentration (LD50) of EMS for generating the mutagenized population was determined by treating seeds with different concentrations of EMS (0.0, 0.2, 0.4, 0.6, and 0.8% v/v) and observing the germination count after 5 days of planting the seeds in Petri dishes. Three thousand cowpea seeds were treated with the 0.4% EMS to generate the M_1_ and M_2_ populations that were evaluated for agronomic and morphological traits with untreated seeds serving as control. Data analysis involved distribution of qualitative and quantitative traits. Germination was significantly reduced in the mutagenized population (17.8%) and compared with that of the wild type (61.6%). Percentage survival was significantly higher in wild type (98.38%) as compared with the M_1_ population (78.46%). Percentage germination in the M_2_ population (74.03%) was lower than the wild type (80%). A wide spectrum of agro-morphological abnormalities was observed in the M_2_ population. Wide variations and uniquely different phenotypic classes were observed in leaf color, leaf shape, growth habit, plant pigmentation, twining tendency, pod curvature, seed shape, and seed coat color. M_2_ individuals were widely distributed for days to flowering, number of pods per plant, number of seeds per pod, number of locules per pods, percentage seed set, pod length and number of seeds per plant. In conclusion, the EMS mutagenesis was effective in inducing the unique variations that will be useful for breeding and development of new farmer preferred varieties.

## Introduction

Induced mutagenesis has been recognized recently as an important supplement to conventional breeding in crop improvement programs. Since the prominent discoveries by Muller (1927) and Stadler (1928a,b), large amount of genetic variability has been induced by various mutagens. The use of induced mutation over the years has contributed to modern plant breeding programs. About 3,500 varieties have been released around the globe through mutation breeding (IAEA, 2021). The majority of mutants released all over the world are food crops (IAEA, 2021). Among the varieties released, very few are pulses. About 180 mutant varieties of soybean have been released and 10 mutant varieties of cowpea released in India. In Africa no variety of cowpea has been developed through mutagenesis (IAEA, 2021). The application of mutagenesis in breeding programs in Ghana has led to the development of two mutant varieties in two crops; *Manihot esculanta*, Crantz (high yielding cassava mutant with 40% dry matter content), and *Theobromo caocao* (a cocoa swollen shoot disease resistant mutant variety) (Danso et al., 2008).

In the genetic improvement of crops, molecular plant breeders have the option of studying natural variations or induced variations. The induction of mutations involves the artificial processes of subjecting an organism (plant, animal, microbes, or any living system) to physical and chemical mutagens in other to create variations at the molecular level which can be observed as physical characters and may be stably inherited. Chemical mutagens such alkylating agents (ethyl methane sulfonate and methyl methane sulfonate), colchicine, sodium azide as well as physical mutagens, such as ionizing radiations have been used to increase the frequency of variations and mutations in organism (Predieri, 2001). Chemical mutagens are more efficient as compared to the physical mutagens. This is because chemical mutagens such alkylating agents cause point mutation which mostly results in a base pair change (GC → AT change) which are trackable and therefore useful in gene mapping and trait phenotype association studies. The change in base pair may affect expression of gene resulting to variations in characters of traits. Physical mutagens on the other hand cause deletions of part of DNA or chromosome. The random deletions caused by physical mutagen may result in deleting the gene of desirable traits thereby abolishing the function of the gene. According to Bhat et al. (2005), chemical mutagens enhance genetic variability in plants for successful breeding programs. Previous studies by Adamu and Aliyu (2007) reported that chemical mutagen induced a wide range of variation in morphological traits when compared with normal plants.

Ethyl methane sulfonate has been highly effective and efficient in producing diversity in agronomic traits of different varieties of legumes including cowpea. The efficiency of an EMS mutagen describes the rate at which the mutagen is able to induce changes in the genetic material creating a range of desirable and undesirable effects. Various mutagens including EMS are known to induce morphological mutation such as chlorophyll mutation and viable mutations in crops such as cowpea, pepper, and mung bean (Gnanamurthy and Dhanavel, 2014). Induced mutation has an effect on the lethality and germination rates, chlorophyll content, viability, and other factors adding up to the improvement on the morpho-agronomic nature of cowpea plant (Dhanavel et al., 2008; Dhanavel and Girija, 2009). Bind and Dwivedi (2014), in their study reported decrease in percentage germination, percentage plant survival and increase in pollen sterility in cowpea treated with doses of EMS.

Various concentrations of the chemical EMS produce wide range of mutations which are used to determine mutation frequency. The frequency of viable mutations will depend on the treatment conditions. The dose of EMS concentration and time taken have impact on the viability of mutations produced by EMS. In order to produce a high frequency of desirable mutations in a crop, it is very important to determine the lethal dose 50 (LD50) (Hohmann et al., 2005; Arisha et al., 2014). Nair and Mehta (2014) reported highest mutation frequency of 1.58% in cowpea treated with 0.35% EMS. LD50 is the EMS concentration that contributes to the 50% lethality of the total number of seeds or propagules that is subjected to the mutagenesis treatment.

The functional mutations in genes induced by EMS as a result of point mutation have a greater chance of being dominant or co-dominant mutations. During the M_1_ generation, only dominant or co-dominant mutations are easily detected by phenotypic observations (Shu et al., 2012). Some viable mutations in the M_1_ generation include reduction in plant height, pollen sterility, late or early flowering, leaf chlorosis, and curled leaves, occurrence of irregular leaf structure. During the M_2_ generation, there is segregation of alleles which leads to homozygotes for recessive and dominant alleles thereby creating the opportunity to identify the recessive mutations *via* phenotyping (Page and Grossniklaus, 2002). Here, the objective was to chemically induce mutations to create unique genetic variations in the cowpea cultivar, Asontem and characterize them for breeding purposes.

## Materials and methods

### Experimental materials

Cowpea variety (“*Asontem*”) was used for the study. IT82E-16 (“*Asontem*”) is a cultivar that was developed by International Institute for Tropical Agriculture (IITA), and released by Council for Scientific and Industrial Research – Crops Research Institute, Ghana. “*Asontem*” has indeterminate growth pattern with days to maturity ranging from 70 to 76. The leaves are narrow (hastate) and the growth habit is intermediate. The *Asontem* seeds were obtained from the West Africa Center for Crop Improvement, University of Ghana (WACCI) gene bank.

### Experimental procedure

#### Sensitivity test for determination of lethal dose 50

Cowpea seeds were mutagenized with different EMS concentrations (0.0, 0.2, 0.4, 0.6, and 0.8%). Each concentration had 100 “*Asontem*” seeds. Seeds were soaked in the solutions for 16 h. After that, the chemical reaction and activity of the EMS was terminated using sodium thiosulfate solution to neutralize and stop the reaction by inactivating the EMS. The treated seeds were plated in Petri dishes and number of seeds germinated were observed after 5 days.

#### Generation of M_1_ seeds

Three thousand cowpea (*Asontem*) seeds were treated with 0.4% EMS concentration. The viability of the seeds was generally low, however, obtaining new Asontem seeds was difficult so the available seeds were used for this experiment. The 0.4% concentration was prepared by adding 2.2 ml of EMS to 547.8 ml of distilled water. M_1_ seeds were obtained after soaking seeds in EMS concentration for 16 h based on the protocol for EMS mutagenesis. Five hundred seeds were soaked in 0.0% EMS concentration (distilled water) which served as control (Wild type).

#### Experimental layout and field establishment

The wild type (non-treated seeds) and mutagenized seeds were sown on the university of Ghana farms in a single row plot design. This area is coastal savannah zone of the country’s agroecological zones. Amount of rainfall in a year is about 900 mm. A total of nineteen rows were established for the study; sixteen rows comprised the mutagenized seeds and three rows were made of the control (which served as wild type). Seeds were sown at three seeds per hill with four rows of mutant plants and one row of wild type in a sequential manner. The planting distance used was 0.75 m inter-row and 0.3 m intra- row. A total of sixteen rows and three rows were designated to the mutagenized population and wild type respectively. Wild type seedlings were thinned out to one plant per hill in the fourth week after planting. Agronomic practices such as weed control, irrigation and pest control were carried out in the field.

During the evaluation of the M_2_ generation, 319 mutant plants (M_1_ parents) were used and twenty seeds were obtained from each mutant line. A total of 6,380 M_2_ seeds and 100 wild type seeds were used. A total of three hundred and twenty-four (324) rows were established for the study; three hundred and nineteen (319) rows comprised the M_2_ plants and five (5) rows were made of the wild type. The field was divided into five (5) blocks and each block was made up of 65 rows. Each block had 64 rows of M_2_ plants and one row of the wild type except the last block which comprised 63 rows of M_2_ plants and one row of wild type. The rows were made up of twenty seeds from each mutant line (parent). Seeds were sown at one seed per hill. The planting distance used was 0.75 m inter-row and 0.3 m intra- row. The rows in each block were randomized to contain the mutant plants and the wild type. A total of three hundred and nineteen rows and five rows were designated to M_2_ plants and control respectively. Agronomic practices such weed control, irrigation and pest control were carried out.

### Morpho-agronomic characterization of M_1_ and M_2_ generations

#### Parameters studied in M_1_ generation

The morphological and agronomic characteristics studied were quantitative and qualitative traits. Quantitative and qualitative data on the following traits were collected based on the Cowpea Descriptor by International Board of Plant Genetic Resources (IBPGR) (1983).

Qualitative data collected were: plant pigmentation, growth habit, flower color, terminal leaflet shape, pod shape/curvature, pod color, seed shape and seed coat color. Plant pigmentation, growth habit, flower color and terminal leaflet shape were determined during the flowering stage and pod shape/curvature, pod color, seed shape, and seed coat color were also observed after harvesting.

Quantitative data collected comprised: chlorophyll content, days to flowering, days to first pod maturing, number of pods per plant, pod length, number of locules per pod, number of seeds per pod. Data on days to flowering was observed on the first day of flowering. Number of pods per plant, pod length, number of locules per pod, number of seeds per pod were observed after harvesting.

#### Parameters studied in M_2_ generation

Qualitative data collected and analyzed were: terminal leaflet shape, plant pigmentation, leaf marking, growth habit, leaf color twinning tendency growth pattern, pod shape/curvature, pod color, seed coat color, and seed shape. Data on terminal leaflet shape, plant pigmentation, leaf marking, growth habit, leaf color twinning tendency growth pattern were obtained during flowering stage. Pod shape/curvature, pod color, seed coat color, and seed shape were observed after harvesting.

Quantitative data collected and analyzed comprised: percentage germination, percentage survival, germination speed, days to flowering, number of pods per plant, pod length, number of locules per pod, number of seeds per pod, percentage seed set, and number of seeds per plant. Percentage germination data were obtained after 15 days of planting. Data on days to flowering was observed on the first day of flowering. Data on number of pods per plant, pod length, number of locules per pod, number of seeds per pod, percentage seed set and number of seeds per plant were collected after harvesting.

Germination percentage was found by observing the emergence of the coleoptile at the surface of the soil. The total number of seeds germinated in each treatment was recorded after 21 days of planting.

Germination percentage was obtained as; % germination = n⁢u⁢m⁢b⁢e⁢r⁢o⁢f⁢s⁢e⁢e⁢d⁢s⁢g⁢e⁢r⁢m⁢i⁢n⁢a⁢t⁢e⁢dt⁢o⁢t⁢a⁢l⁢n⁢u⁢m⁢b⁢e⁢r⁢o⁢f⁢s⁢e⁢e⁢d⁢s⁢p⁢l⁢a⁢n⁢t⁢e⁢d⁢x⁢ 100%

Germination speed was also calculated as described by Maguire (1962): GS = $\sum_{j}^{i}\frac{n\,i}{D\,i}$

*ni* is the number of seeds germinated on the *i*^th^ day and *Di* is the number of days after planting.

Percentage seed set was calculated as; % seed set = n⁢u⁢m⁢b⁢e⁢r⁢o⁢f⁢s⁢e⁢e⁢d⁢s⁢p⁢e⁢r⁢p⁢o⁢dn⁢u⁢m⁢b⁢e⁢r⁢o⁢f⁢l⁢o⁢c⁢u⁢l⁢e⁢s⁢p⁢e⁢r⁢p⁢o⁢d⁢x⁢ 100%

Number of seeds per plant was estimate as; number of seeds per pod *x* number of pods per plant.

### Selection of putative mutants

Regarding each character evaluated, the performance of the wild type was determined. Mutants or plants differing from the wild type plants with regard to each character studied was identified by simply comparing it with that of the control or wild type character. A test of statistical significance was then carried out to highlight the difference.

### Statistical analysis

Frequency distribution of qualitative variables in both the wild type and the mutagenized populations were computed using STATA statistical software version 14 (StataCorp, 2015). Distributions of quantitative traits in the populations were determined using R software version 4.0.1. Excel was used to compute estimates of percentage germination, germination speed and number of seeds per plant. Student *T*-test or Z-test was used to determine significant difference between the wild type and mutants with performances above the control (wild type) range.

## Results

### Sensitivity assay for determination of lethal dose 50

**FIGURE 1:**
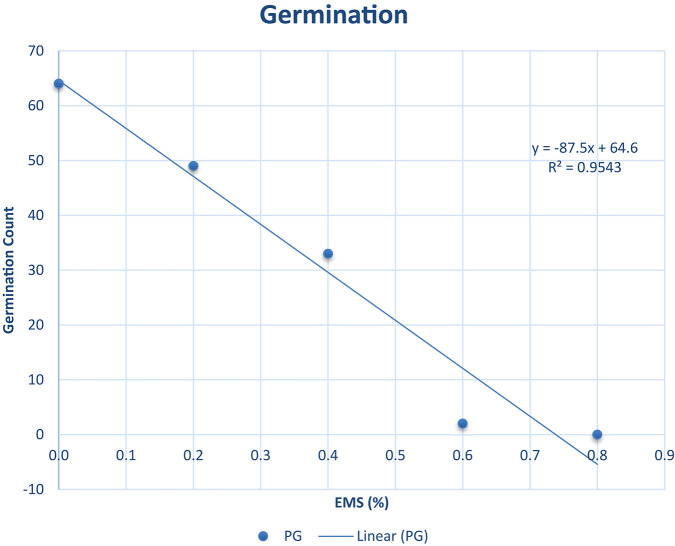
Germination percent (%) and line of best fit for estimation of the LD50 in *Asontem* cowpea genotype when subjected to five EMS doses for 16 h. PG, percentage germination.

The percentage germination ranged from 0.00 to 63.00% as described in Figure 1. It was observed from our experiments that the percentage germination decreased as EMS concentration increased (R^2^ = 0.9543). The highest percentage germination was recorded in the wild type and the lowest was recorded in EMS dose 0.8%. The EMS lethal dose 50 (LD50) was estimated to be approximately 0.4% concentration of EMS solution where 34% germinated. The 34% is approximately 50% of 63 (wild type individuals that germinated). The 63% germination in the wild type was assumed as 100% germination since there was no EMS treatment. Hence, 0.4% EMS was estimated as the LD50 for *Asontem* since this level of EMS concentration caused 50% lethality comparatively with the wild type.

### Percentage germination and survival of M_1_ plants

A total of 534 seeds germinated in the M_1_ population out of the 3,000 seeds sowed after application of 0.4% EMS for 16 h whiles 308 seeds germinated from the 500 wild type seeds (Table 1). The viability of the seeds was low from the sensitivity test. The percentage survivals obtained in M_1_ population, and the wild type were 74.46 and 98.38%, respectively.

**TABLE 1:** Percentage germination and percentage survival in the M_1_ generation.

EMS (%) dose treatment	Number of seeds sowed	No of germinated seeds	Percentage germination	Percentage survival
0.0	500	308	61.6	98.38
0.4	3,000	534	17.8	78.46

### Morpho-agronomic characterization of M_1_ plants

#### Frequency distribution of qualitative traits in M_1_ population

##### Stem pigmentation

**FIGURE 2:**
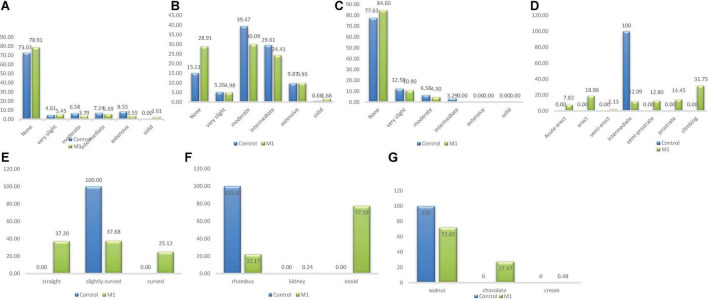
Distribution of qualitative traits among the M_1_ plants and + wild type. **(A)** Stem pigmentation, **(B)** branch pigmentation, **(C)** petiole pigmentation, **(D)** growth habit, **(E)** pod curvature, **(F)** seed shape, **(G)** seed coat color.

There were five categories of stem pigmentation in the wild type population and six categories in the M_1_ population as depicted in Figure 2. The unique stem pigmentation described as solid was clearly absent in the natural control population but present in the induced mutant population. The frequency distribution ranged from 4.61% (*very slight*) to 73.03% (*None*) in the wild type. The highest frequency in the M_1_ population was 78.91% (*None*) and the lowest was 2.61% (*Solid*).

##### Petiole pigmentation

Four different phenotypic classes were observed in the wild type whiles three classes were observed in the M_1_ population (Figure 2). *Intermediate* class which was observed only in the wild type was the lowest (frequency = 3.29%) and *None* class was the highest frequency (77.63%) in the wild type. In the M_1_ population, the frequency ranged from 4.50% (*Moderate*) to 84.60% (*None*). *Intermediate* class was absent in the mutant plants.

##### Branch pigmentation

Branch pigmentation had six different phenotypic categories in both the wild type and M_1_ population. Frequency distribution based on treatment ranged from 0.66% (*Solid*) to 39.47% (*Moderate*) for the wild type, while for M_1_ population the range was between 1.66% (*None*) and 30.09% (*Moderate*) (Figure 2).

##### Growth habit

There was only one category (*intermediate*) identified for this trait in the wild type, whiles seven categories were observed in the M_1_ population. The six different categories that were present in the M_1_ were absent in the wild type. The frequency ranged from 2.13% (*semi-erect*) to 31.75% (*Climbing*) in the M_1_ population (Figure 2).

##### Leaf shape

Only one phenotypic class (*Hastate*) was observed for this trait in both wild type and M_1_ population.

##### Flower color

There was only one phenotypic category (*violet*) identified in both the wild type and M_1_ population.

##### Pod color

Pod color had only one phenotypic class (*Pale tan*) in both the wild type and M_1_ population.

##### Pod curvature

There was one category of pod curvature (*slightly curved*) in the wild type and three categories in the M_1_ population (Figure 2). The highest frequency in the M_1_ population was 37.68% (*slightly curved*) and the lowest was 25.12% (*curved*). The *curved* and *straight* categories of pod curvature were absent in the wild type.

##### Seed shape

Only one phenotypic class (*rhomboid*) was observed in the wild type whiles three classes were observed in the M_1_ population as shown in Figure 2. The different seed shapes that were observed only in the mutant population were *kidney* and *ovoid*. *Kidney* class had the lowest frequency (0.24%) and *ovoid* class had the highest frequency (77.59%) in the M_1_ population.

##### Seed coat color

There was one phenotypic category (*walnut*) that occurred in the wild type whiles three different categories were obtained in the M_1_ population. The different seed coat colors described as *cream* and *chocolate* were observed in the M_1_ plants. Frequency distribution ranged from 0.48% (*cream*) to 72.05% (*walnut*) for the M_1_ population.

#### Distribution of quantitative traits in M_1_ generation

##### Chlorophyll content

**FIGURE 3:**
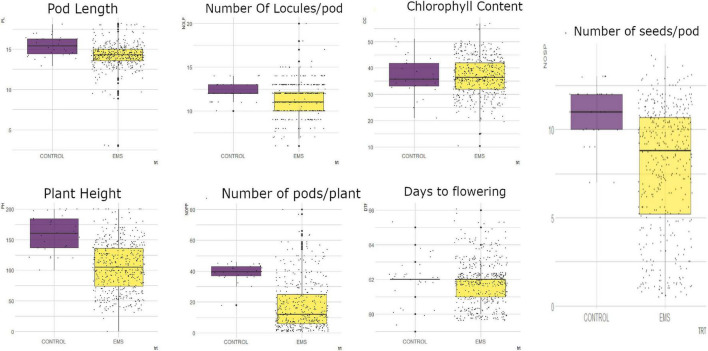
Distribution of quantitative traits studied into 25th percentile, 50th percentile, and 75th percentile in the wild type (control) and mutant population. Black dots represent the dispersion and frequency distribution of the phenotypic data in each treatment. PL, pod length; PH, plant height; NOLP, number of locules per pod; NOPP, number of pods per plant; CC, chlorophyll content, DTF, days to flowering; NOSP, number of seeds per pod; trt, treatment; EMS, ethyl methane sulfonate.

From measurements of the chlorophyll content in the leaves of M_1_ generation, the mutagenized population was widely distributed with some outliers as shown in Figure 3. The wild type plants (*Asontem*) had a range of 20.9–51.00 mg/l for chlorophyll content and the mutagenized population had a range of 10.40–56.90 mg/l. The median value obtained in the mutagenized population (36.45 mg/l) was slightly above the median value recorded in the wild type (35.70 mg/l).

##### Plant height

There was high phenotypic variation for plant height (PH) in the mutagenized population of the M_1_ generation whereas low variation was observed in the wild type (Figure 3). The wild type had a range of 100 cm (from 100 to 200 cm) and the range of the mutagenized population was 180 cm (from 20 to 200 cm). The individuals in the M_1_ population were widely dispersed with a median value of 113 cm whereas 50% of the wild type were above 160.5 cm for plant height.

##### Days to flowering

In the M_1_ generation, there was low phenotypic variation observed in both the wild type and the mutagenized population for days to flowering. There were few outliers in both the treated and the wild type (Figure 3). The mutagenized population ranged from 60 to 66 days and the wild type ranged 59–65 days. A median value of 62 days was obtained in both the M_1_ population and the wild type.

##### Number of pods per plant

In the M_1_ generation, the mutagenized population was widely dispersed whiles the wild type was narrowly dispersed for number of pods per plant as shown in Figure 3. The wild type ranged from 18 to 46 pods per plant and the mutagenized population ranged from 1 to 80 pods per plant. Fifty percent of the mutagenized population had less than 12 pods and the median value in the wild type was 40 pods.

##### Pod length

High phenotypic variation was observed in the mutagenized population with many outliers for pod length (Figure 3). The mutagenized population ranged from 3.0 to 18.20 cm and the wild type ranged from 12.9 to 18.1 cm in the M_1_ generation. The variation in the wild type was low. The median values recorded for this trait in the wild type and the mutagenized population were 15.4 and 14.3 cm, respectively.

##### Number of locules per pod

There were some outliers observed in both the wild type and mutagenized population for number of locules per pod (Figure 3). However, there was high phenotypic variation in the M_1_ population as compared to the wild type. The wild type ranged from 10-14 and the mutagenized population ranged from 6 to 20. Fifty percent of the individuals within the mutagenized population had 11 locules and above whiles the median value for the wild type was 12.

##### Number of seeds per pod

In the M_1_ generation, there was high phenotypic variation in the mutagenized population whereas low variation was observed in the wild type for number of seeds per pod (Figure 3). The range of the wild type was 5 (from 7 to 13) and the range of the mutagenized population was 13 (from 1 to 14). The individuals in the mutagenized population are widely spread with a median value of nine whiles the individuals in the wild type were clustered around the median (11).

### Phenotyping of M_2_ generation

#### Percentage seed germination and germination speed of M_2_ generation

Percentage seed germination ranged from 5 to 100% among the M_2_ plants. Total percentage seed germination recorded in the M_2_ population was 74.03% and the wild type had 80% (Table 2). Percentage survival recorded in the M_2_ population was 95.80% whiles 100% survival was observed in the wild type. Germination speed decreased in the M_2_ population as compared with the wild type. The values recorded were 1.43 and 1.56 in the mutagenized population and the wild type, respectively (Table 2).

**TABLE 2:** Percentage germination, percentage survival and germination speed of the wild type and M_2_ population.

	Seeds sown	Germination count	Percentage germination	Number of surviving plants	Percentage survival	Germination speed
M_2_ population	6,380	4,723	74.03	4,526	95.8	1.43
Wild type	100	80	80	80	100	1.56

#### Morphological mutations in M_2_ population

**FIGURE 4:**
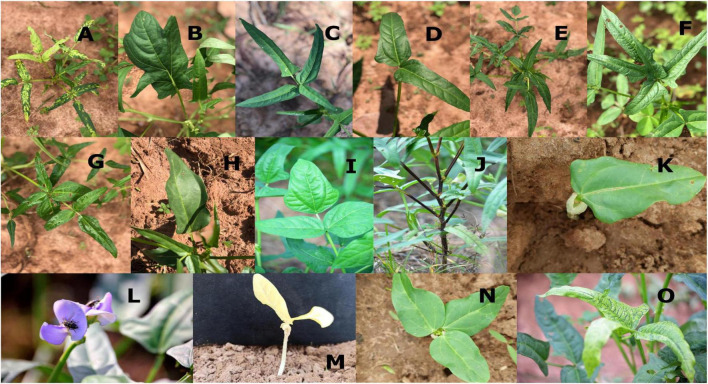
Different morphological mutations in the M_2_ population. **(A)** Variegated leaf plant, **(B)** irregular leaf, **(C)** tetrafoliate leaf, **(D)** bifoliate leaf, **(E)** hexafoliate leaf, **(F)** pentafoliate, **(G)** septafoliate, **(H)** monofoliate, **(I)** sub-hastate leaf, **(J)** solid pigmented plant, **(K)** monopinnate leaf, **(L)** violet flower, **(M)** xantha seedling, **(N)** tripinnate leaf, **(O)** variegated leaf.

A wide range of morphological mutations were observed in the M2 population (Figure 4). There were thirty (30) individuals that showed chromosomal mutations in 4,526 individuals observed in the M_2_ population (Table 3). There were eighteen plants that showed variegated leaves (frequency = 0.04), three plants were albino (frequency = 0.07). There were six plants that showed yellow single leaf (0.13) and three plants were xantha (frequency = 0.07).

**TABLE 3:** Frequency of individuals with morphological abnormalities in the mutagenized population (M_2_ generation).

Abnormalities	Number of plants	Frequency (%)
Monofoliate	3	0.07
Bifoliate	39	0.86
Tetrafoliate	22	0.49
Pentafoliate	6	0.13
Hexafoliate	1	0.02
Septafoliate	1	0.02
Variegated leaf	18	0.40
Albino	3	0.07
Xantha	3	0.07
Irregular leaves	24	0.53
Single globose leaf	1	0.02
Single yellow leaf	6	0.13
Single pinnate leaf	1	0.02
Triple pinnate leaves	5	0.11

A wide spectrum of leaf mutations was observed in the mutant population with noticeable variation in size, shape, number and arrangement of leaflets (Table 3). Some mutants have irregular leaves. These mutants were described by the presence of leaves with serrated leaves, irregular leaf margins, abnormal vernation and irregular shape of lamina.

Individuals with abnormal leaflet numbers were observed in the M_2_ population. These mutants produced leaves with less than three or more leaflets. There were mutants with single leaflet, bifoliate (two leaflets), tetrafoliate (four leaflets), pentafoliate (five leaflets), hexafoliate (six leaflets), and septafoliate (seven leaflets).

#### Frequency distribution of qualitative traits among wild type and M_2_ population

##### Leaf color

**FIGURE 5:**
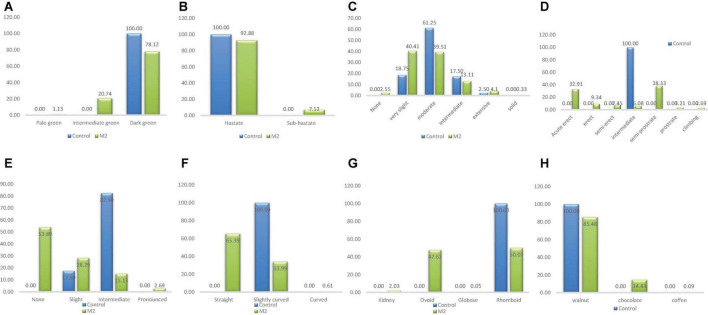
Distribution of qualitative traits among the M_2_ plants and + wild type. **(A)** Leaf color, **(B)** leaf shape, **(C)** plant pigmentation, **(D)** growth habit, **(E)** twinning tendency, **(F)** pod curvature, **(G)** seed shape, **(H)** seed coat color.

**FIGURE 6:**
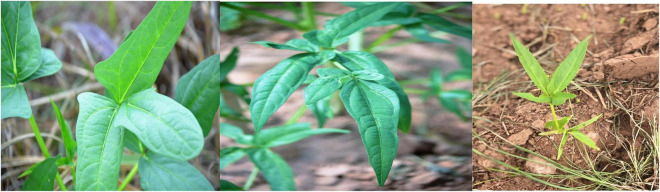
Different leaf colors observed in the mutant population.

There was one phenotypic class (*dark green*) of leaf color in the wild type and three categories in the M_2_ population (Figure 5). *Pale green* and *intermediate green* leaf colors were the different classes that were observed only in M_2_ population (Figure 6). The frequency ranged from 1.13% (*pale green*) to 78.12% (*dark green*) in the M_2_ population.

##### Leaf shape

One phenotypic class (*Hastate*) was observed in the wild type whiles two classes were observed in the M_2_ population (Figure 5). *Sub-hastate* class which was clearly absent from the wild type plants had the lowest frequency (7.12%) and *Hastate* class had the highest frequency (92.88%) in the M_2_ population.

##### Plant pigmentation

There were four different phenotypic categories in the wild type and six different classes in the M_2_ population as shown in Figure 5. The unique class (solid pigmentation) which was only present in the M_1_ plants was also observed in the M_2_ plants. The different category described as *none* was present in the M_2_ plants but it was clearly absent in the wild types. The highest frequencies recorded based on treatment were 61.25% (*Moderate*) and 40.41% (*Very slight*) for the wild type and M_2_ population respectively. The lowest frequency observed in the wild type was 2.5% (*Extensive*) whiles 0.33% (*Solid*) was the lowest frequency observed in the M_2_ population.

##### Growth habit

This trait had only one phenotypic class (*Intermediate*) in the wild type and seven categories in the M_2_ population which is depicted in Figure 5. Same classes of growth habit that were observed in the M_1_ plants were observed in the M_2_ plants. The frequency ranged from 2.69% (*Cimbing*) to 38.33% (*Semi-prostrate*) in the M_2_ population.

##### Leaf marking

There was only one phenotypic class (*present*) identified in both the wild type and M_2_ population.

##### Growth pattern

*Indeterminate* growth pattern was the only phenotypic class observed in both the wild type and M_2_ population.

##### Flower color

There was only one phenotypic class observed for these traits in the wild type and the M_2_ population.

##### Pod color

There was only one phenotypic class (*pale tan*) observed in both the wild type and M_2_ population.

##### Twinning tendency

There were two different phenotypic categories in the wild type and four different classes in the M_2_ population (Figure 5). The categories; *none* and *pronounced* were absent from the wild type. The highest frequencies recorded based on treatment were 82.50% (*intermediate*) and 53.89% (*None*) for the wild type and M_2_ population respectively. The lowest frequency observed in the wild type was 17.50% (*Slight*) whiles 2.69% (*Pronounced*) was the lowest frequency observed in the M_2_ population.

##### Pod curvature

One phenotypic class (*Slightly curved*) was observed in the wild type whiles three classes were observed in the M_2_ population (Figure 5). *Curved* class had the lowest frequency (0.61%) and *Straight* class had the highest frequency (65.39%) in the M_2_ population.

##### Seed shape

Only one phenotypic class (*rhomboid*) was observed in the wild type whiles three classes were observed in the M_2_ population as shown in Figure 5. The different seed shapes that were observed only in the mutant population were *kidney, globose* and *ovoid*. *Globose* class had the lowest frequency (0.05%) and *rhomboid* class had the highest frequency (50.07%) in the M_2_ population.

##### Seed coat color

There was one phenotypic category (*walnut*) that occurred in the wild type whiles three different categories were obtained in the M_2_ population as shown in Figure 5. The different seed coat colors described as *coffee* and *chocolate* were observed in the M_1_ plants and as well as the M_2_ plants. Frequency distribution ranged from 0.09% (*coffee*) to 85.48% (*walnut*) for the M_2_ population.

#### Distribution of yield and sub-yield characters of the M_2_ generation

Data was collected on a total of 2,201 individual plants at the M_2_ generation; 2,121 mutagenized individuals and 80 individuals in the wild type.

##### Days to flowering

**FIGURE 7:**
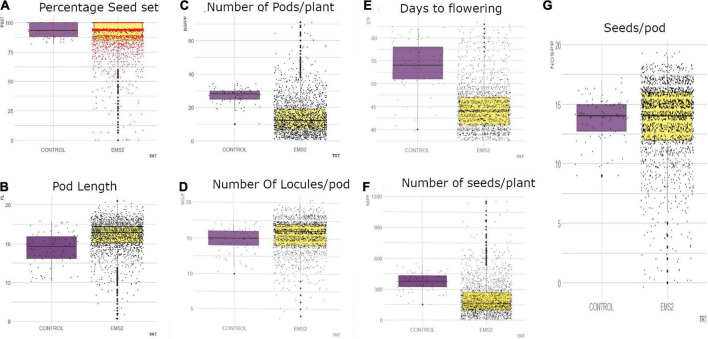
Distribution of quantitative traits studied in M_2_ generation into 25th percentile, 50th percentile and 75th percentile in the wild type (control) and mutant population. Black dots represent the dispersion and frequency distribution of the phenotypic data in each treatment. **(A)** Boxplot for percentage seed set (PSST), **(B)** Boxplot for pod length (PL), **(C)** Boxplot for number of pods per plant (NOPP), **(D)** Boxplot for number of locules per (NOLP), **(E)** Boxplot for Days to flowering (DTF), **(F)** Number of seeds per plant, **(G)** Number of seeds per pod (NOSP), trt, treatment, EMS, ethyl methane sulfonate.

In the M_2_ generation, the wild type and M_2_ population showed continuous phenotypic variation for days to flowering (Figure 7). The wild type ranged from 40 to 62 days and the mutagenized population ranged from 38 to 63 days. The median value obtained in the wild type was 54 and the mutagenized population’s median value was 44. Fifty percent of the individuals in the mutant population flowered within 44 days.

##### Number of pods per plant

There was high phenotypic variation in the mutagenized population with some outliers whereas low variation was observed in the wild type for number of pods per plant in the M_2_ generation (Figure 7). The range of the mutagenized population was 70 pods (from 1 to 71 pods) and the range of the wild type was 14 pods (from 10 to 34 pods). The median value obtained in the wild type was 28 and the median value recorded in the M_2_ population was 12. Fifty percent of the mutagenized population had 1–12 pods.

##### Number of seeds per pod

The mutagenized population in the M_2_ generation was widely distributed whiles the wild type was narrowly distributed for number of seeds per pod as shown in Figure 7. There were outliers observed in both the wild type and mutant population. The wild type ranged from 9 to 17 seeds per pod and the mutagenized population ranged from 0 to 19 seeds per pod. The median value obtained in both the wild type and the M_2_ population was 14 seeds per pod.

##### Number of locules per pod

In the M_2_ generation, there were some outliers observed in both the wild type and mutagenized population for number of locules per pod (Figure 7). However, there was high phenotypic variation in the mutagenized population as compared to the wild type for this trait. The wild type ranged from 10-17 locules per pod and the mutagenized population recorded a range of 4–20 locules per pod. The median value obtained in the M_2_ population (median = 16) was higher as compared to the wild type (median = 15). Fifty percent of the mutant population had 16 and more locules in a pod.

##### Percentage seed set per pod

The mutagenized population had high phenotypic variation with many outliers for percentage seed set per pod (Figure 7). A range of 81.82–100% was observed in the wild type and the mutagenized population had a range of 0.00–100%. The median value obtained for both the wild type and the M_2_ population was 93.33%. Fifty percent of the mutagenized individuals in the M_2_ generation had 0 to 93.33% seed set.

##### Pod length

The M_2_ population was widely distributed with many outliers for pod length as shown in Figure 7. The wild type was however narrowly distributed with a range of 12.1–18.3 cm. The mutagenized population had a range of 8.3–20.4 cm. Fifty percent of the individuals in the M_2_ population had pod length of 17.1 cm and above. The median value recorded in the wild type was 15.7 cm.

##### Number of seeds per plant

In the M_2_ generation, high phenotypic variation was observed in the mutagenized population with many outliers for number of seeds per plant (Figure 7). The mutagenized population ranged from 0 to 1,156 and the wild type ranged from 150 to 544. The variation in the wild type was low. However, the median value obtained in the wild type was higher than median value recorded for the mutant population. Fifty percent of individuals in the M_2_ population had 0 to 165 numbers of seeds.

#### Putative mutants that showed high performance above the wild type in the M2 generation

Individuals in the M_2_ population that showed higher performance for the quantitative traits than the wild type were selected as putative mutants. Days to flowering had the highest number of individuals that showed high performance above the wild type (z = 19.82, *p* < 0.05) with frequency of 27.06% (Table 4). There were eighty-five (85) individuals in the mutagenized population that exhibited higher performance above the wild type (z = 16.88, *p* < 0.05) with the least frequency (4.01%) for number of seeds per plant.

**TABLE 4:** Frequency of selected putative mutants from 2,121 M_2_ individuals showing high performance above the wild type for six (6) quantitative traits.

Traits	Number of individuals	Frequency (%)	z-statistic
NOPPP	101	4.8	15.58
NOSPP	86	4.05	20.3
NOLP	290	13.67	18.64
PL	231	10.89	17.31
DTF	574	27.06	19.85
NSPP	85	4.01	16.88

#### Putative mutants that showed lower performance than the wild type in the M_2_ generation

There were 797 individuals out of the 2,121 individuals observed that exhibited significantly lower performance below the wild type (z = 15.58, *p* < 0.05) for number of pods per plant (Table 5). The highest frequency of putative mutants was observed in number of pods per plant. Days to flowering had the least frequency (0.80%) of individuals that performed significantly lower than the wild type (z = 23.39, *p* < 0.05).

**TABLE 5:** Frequency of selected putative mutants from 2,121 M_2_ individuals showing lower performance than the wild type for six (6) quantitative traits.

Traits	Number of individuals	Frequency (%)	z-Statistic
NOPPP	797	37.58	42.84
NOSPP	125	5.89	26.59
NOLP	38	1.79	22.47
PSST	426	20.08	24.57
PL	25	1.18	15.62
DTF	17	0.80	23.39

NOPPP, number of pod per plant; NSPP, number of seeds per plant, PL, pod length; NOLP, number of locules per pods; NOSPP, number of seeds per pod; PSST, percentage seed set; DTF, days to flowering.

#### Contrast analysis

Some of the selected individuals in the mutagenized population showed significant difference (*p* < 0.01) for seven (7) quantitative traits observed when compared with wild type mean. For individuals that performed higher than the wild type, there were 55 that were significantly different from the wild type for number of pods per plant; 8 were significantly different from wild type for number of seeds per pod (Table 6). There was no individual that showed significant different from the wild type mean for percentage seed set.

**TABLE 6:** Frequency of highly significant mutants from 2,121 M2 individuals showing high performance above the wild type for six (6) quantitative traits.

Traits	Number of plants	Frequency	*P*-value
NOPPP	55	2.59	<0.01
NOSPP	8	0.38	<0.01
NSPP	61	2.88	<0.01
NOLP	38	1.79	<0.01
PSST	0	0	<0.01
PL	11	0.52	<0.01
DTF	251	11.83	<0.01

NOPPP, number of pod per plant; NSPP, number of seeds per plant; PL, pod length; NOLP, number of locules per pods; NOSPP, number of seeds per pod; PSST, percentage seed set; DTF, days to flowering.

For individuals that performed lower below the wild type range, there were 795 that showed significant difference from the wild type mean for number of pods per plant; 3 were significantly different from the wild type mean for pod length. There was no individual in the mutagenized population that showed low significant difference for days to flowering (Table 7).

**TABLE 7:** Frequency of highly significant mutants from 2,121 M_2_ individuals showing low performance below the wild type for six (6) quantitative traits.

Traits	Number of plants	Frequency	*P*-value
NOPPP	795	37.48	<0.01
NOSPP	125	5.89	<0.01
NSPP	950	44.79	<0.01
NOLP	7	0.33	<0.01
PSST	296	13.96	<0.01
PL	3	0.14	<0.01
DTF	0	0.00	<0.01

NOPPP, number of pod per plant; NSPP, number of seeds per plant; PL, pod length; NOLP, number of locules per pods; NOSPP, number of seeds per pod; PSST, percentage seed set; DTF, days to flowering.

## Discussion

### Sensitivity test

Determination of mutagenic sensitivity of germinating seeds constitutes an important aspect in mutation breeding, since healthy crop growth and yield depends upon the seedling establishment (Nair and Mehta, 2014). The selection of an effective and efficient mutagen concentration and growth condition is essential to produce a high frequency of desirable mutations in mutation breeding programs (Wani, 2009; Arisha et al., 2014). The current study showed that percentage seed germination decreased as concentration of ethyl methane sulfonate increased for the determination of LD50. The wild type had the highest percentage seed germination (63.0%) whereas mutagenic treatment 0.8% had the lowest percentage seed germination (0.0%). The decrease in percentage germination in the treated could be due to the effect of EMS. The LD50 for this study was 0.4%. This is in contrast with the report by Sahrish et al. (2019) in cowpea and Arisha et al. (2015) in pepper. LD50 is genotype specific and it also depends on the treatment time. The different results obtained in this study could be due to the treatment time and the genotype. Alcantara et al. (1996) reported similar results in pepper. The 63% germination obtained in the 0.0% treatment could be as a result of decrease in seed viability of the *Asontem* seeds. This could be due to over storage or seeds not properly stored.

In comparison to the wild type, the percent germination was low in all mutant populations (M_1_ and M_2_ populations). Similar results were reported by Ramya et al. (2014), Bind and Dwivedi (2014), and Uma and Salimath (2001). Significantly reduced germination percentage due to EMS treatment has also been reported in study by Shah et al. (2008) in chick pea. Reduction in percentage seed germination in EMS treated M_*o*_ seeds in Cucumber (*Cucumis sativus* L.) was reported by Wang et al. (2014). The percentage seed germination decrease in EMS treated populations may be resulted from physiological and acute chromosomal damage (Gaur et al., 2003; Nawale et al., 2006). According to Bind and Dwivedi (2014), delay in the one set of mitosis and chromosomal aberration induced enzyme activity such as catalase, lipase and hormonal activity results in reduced germination. Also, the reduction in germination may be due to the seeds absorbing the mutagen, which subsequently reaches the meristemic region and affects the germ cell (Serrat et al., 2014).

Germination speed is very important in cowpea cultivation. Germination speed decreased in the mutagenized population in this study. Ariramana et al. (2014) reported similar results in pigeon pea. This result is in agreement with earlier report by Baghery et al. (2016) in okra. The EMS cause random point mutations as Sikora et al. (2011) proposed. As much as the concentration of EMS increases, the probability of point mutation induction would be increased. These mutations may lead to defects in the synthesis of essential compounds (hormones) for the plant. The EMS might have caused changes in the synthesis and regulation of abscisic acid (ABS) and gibberellic acid (GA); hormones responsible for seed dormancy and germination. The decrease in germination speed may be due to decrease in the production of GA and increase in ABS. According to Baghery et al. (2016), the higher doses of EMS probably would cause more genetic injuries on treated plants which may explain why survival rates and germination speed are lower among them. Baghery et al. (2016) reported lowest percentage germination (55.67) in the highest dose (1.05%) and the highest percentage germination (97.33) in the control and the lowest dose (0.55%).

Percentage plant survival (at flowering) in the M_1_ and M_2_ generations varied within the wild type and the mutagenized populations. The wild type had the highest percentage plant survival and the M_1_ population had the lowest. These outcomes are in close agreement with the earlier reports of Nawale et al. (2006), Ugorji et al. (2012), and Dhanavel and Girija (2009). Bind and Dwivedi (2014) also reported similar findings in cowpea. The decrease in plant survival within the M_1_ population may be due to the effect of the EMS. The chemical mutagen might have caused damages in the genes responsible for growth hormone synthesis pathways.

### Morphological variations in qualitative traits of mutant populations

Mutation induction with chemical mutagen like EMS produces functional mutations with a high probability of producing dominant traits in the M_1_ generation which can be inherited in the next mutant generation (Shu et al., 2012; Arisha et al., 2015). Induced mutation has shown impressive results in crop improvement of diverse crops. Mutation induction is the possible means to provide increased levels of variability among crop species thus obtaining variability in limited traits of a particular genotype (Nair and Mehta, 2014). In the study, variations in qualitative and quantitative traits were observed in the mutant populations.

According to Khan et al. (2015), the use of morphological markers plays an important role in germplasm characterization and evaluation, though some morphological traits are influenced by environmental conditions and variation in development stages. Morphological markers can be grouped into two categories; qualitative and quantitative markers. Many cowpea accessions and varieties have been characterized by most researchers by using these markers. In this current research, the qualitative morphological markers used were leaf marking, leaf shape, leaf color, flower color, twinning tendency, growth habit, growth pattern, plant pigmentation, pod color, pod curvature, seed shape, and seed coat color.

Morphological mutants are very important in mutation breeding programs. They play a crucial role in changing the features of a cultivar to improve on it. In the present study some individuals in the M_1_ and M_2_ populations showed morphological abnormalities in growth habit, leaf, flower, pod and seed coat and seed shape. This research had one category of leaf color (dark green) in both the wild type and mutant population in the M_1_ generation; however, three categories were present in the M_2_ population. Whilst all the wild type plants (wild type) had dark green leaves in the second generation, there were individuals with dark green leaves, intermediate green leaves and some individuals with pale green leaves in the M_2_ population. Similar results were observed in the studies conducted by Arisha et al. (2015) when pepper was treated with chemical mutagen (EMS). This may be that the chemical mutagen induced mutation in the DNA sequences of the genes responsible for this trait which did not express at the first generation. Transposable elements could be the reason for the expression and visibility of this variability in leaf color in the M_2_ population.

Leaf v-markings were present in all individuals in both the wild type and mutagenized population. This may due to the fact that the chemical did not cause changes in the genes of this trait.

From the results obtained in the current study, all the plants in the wild type and mutagenized population in the M_1_ generation had *hastate* leaf shapes. However, some individuals in the M_2_ population had different leaf shapes (*sub-hastate*). This is incongruent with the report by Arisha et al. (2015) in pepper. Nair and Mehta (2014) also reported similar results in cowpea treated with EMS. This could be as results of EMS induced mutations at the M_2_ population.

During the M_1_ generation, there was no physiological changes in flower color and pod color. This is in disagreement with the reports by Gnanamurthy and Dhanavel (2014) and Nair and Mehta (2014) in cowpea. Similar observations also occurred in the M_2_ generation. Genetically, during the M_1_ generation the probability of the occurrence of phenotypic mutation is extremely low and only dominant mutations can be identified (Roychowdhury and Tah, 2013). The results obtained in this research may be due to the fact that the genes responsible for the synthesis of flower and pod pigments may be dominant or oligogene and that EMS did not induce mutations in these genes in this case.

According to Egbadzor et al. (2017), growth habit exhibited by a plant would help identify the suitable planting system to use during production. The current investigation showed variations in growth habit in the M_1_ population with *climbing* occurring the most and *semi-erect* occurring the least. During the M_2_ generation too, there was variability in growth habit. Similar results were reported by Nair and Mehta (2014) in cowpea, Arisha et al. (2015) in pepper. According to Matos Filho et al. (2014); Ribeiro et al. (2014), and Lackyan and Dalvi (2015), growth habit in cowpea is monogenic. The results from this study indicates that EMS might have induced mutation in M_0_ seeds when subjected to the chemical mutagen. Mutation induction with chemical mutagen like EMS produces functional mutations with a high probability of producing dominant traits in the M1 generation which can be inherited in the next mutant generation (Shu et al., 2012; Arisha et al., 2015).

Although the breeder may not be interested in traits such as plant pigmentation, morphological markers like this play important role in the characterization and evaluation of cowpea germplasm. From the results obtained in the current investigation, varying forms of plant pigmentation were observed in both the wild type and the mutagenized populations. However, there were only five classes of this trait observed in the wild type and all the phenotypic classes were observed in the mutant population. The frequency ranged from 2.5% (*extensive* plant pigmentation) to 61.25% (*moderate* pigmentation) in the wild type whiles phenotypic class *solid* had 0.32% (lowest) and *very slight* had 40.41% (highest) in the mutant population. Solid pigmented plant had occurred in both mutant generations whiles absent in the wild type. Padi (2003) studied pigmentation in cowpea and reported that the presence of pigment is dominant over the absence of pigment. According to Asante (1991), pigmentation on plant part is found to be a monogenic character. This research also showed different classes of twinning tendency in the wild type and the mutagenized population during the M_2_ generation. All the phenotypic classes for twinning tendency were observed in the mutant population whereas only two classes were observed in the wild type. The occurrence of different phenotypic classes in the mutagenized population and the difference in frequencies may be due to the effect of the chemical mutagen. Alkylating agents such as EMS induce chemical modification of nucleotides, which result in mispairing and base changes which leads to the alteration of gene codons thus, expressing some physical changes.

In the current work, there were different forms of pod curvature observed in the M_1_ generation which continued in the M_2_ generation. This is in concordance with the earlier report by Nair and Mehta (2014) in cowpea. This may be as a result of EMS causing mutations in gene responsible in this trait. The mutation induced in this trait may be dominant mutation.

Seed coat color and seed coat pattern are consumer traits which are consciously or unconsciously under selection by either farmers or consumers. Moh (1971) reported that people in different locations have different preferences in relation to seed coat color of legumes thus, seed coat color is seen as a very essential agronomic character that determines the marketability of a grain legume including cowpea. In the present study, it was observed that different forms of seed coat color were observed in the M_1_ and M_2_ populations. walnut, chocolate, cream, and coffee seed coats were isolated in the mutant populations. Gnanamurthy and Dhanavel (2014) reported similar results by isolating brown and white seed coat colors in cowpea treated with EMS. This may be due to the effect of the chemical mutagen causing mutation in the gene resulting in the changes from dominant pigmentation factor to its recessive form.

During the M_1_ generation, varying forms of seed shapes (*kidney and* ovoid) were observed. This mutation was also observed in the M_2_ population with kidney, ovoid and globose seed shapes observed. A study performed earlier affirms that different forms of seed shape were found in chick pea mutagenized with EMS (Wani and Anis, 2008). Chemical mutagens induce physiological damages, gene mutations and chromosomal aberration in mutagenized individuals which can be observed and measured from seed germination or emergence of seedlings, survival reduction, plant height reduction and fertility reduction or sterility (reduction in pod and seed formation) (Kumar et al., 2009).

### Morphological abnormalities and chlorophyll mutations in M_2_ population

Leaf abnormalities and chlorophyll mutations were observed in the present research. There were mutants with abnormal leaflet number, irregular leaves and leaf variegation. Leaf variegation is a common mutation which can be either a nuclear or cytoplasmic mutation. EMS may have a high specificity for mitochondrial and plastid genomes (Miller et al., 1984). Redei et al. (1984) reported that many plastid mutations interfere with the development of the photosynthetic apparatus. Dhanavel and Girija (2009) and Nair and Mehta (2014) reported leaf mutant such as tetrafoliate leaf and pentafoliate leaf in cowpea treated with EMS. Tetrafoliate leaf was also observed in studies conducted by Auti and Apparao (2009) in mung bean. Gunckel and Sparrow (1961) explained that the leaf abnormalities might be attributed to chromosomal breakage, disrupted auxin synthesis and transport, disruption of mineral metabolism.

Chlorophyll mutations usually show different forms of leaf coloration at seedling stage, also referred to as leaf coloration. It serves as the point of reference for measuring how effective and efficient a mutagen is capable of inducing different forms of mutations resulting in the formation of either desirable or undesirable traits in a particular plant (Devmani et al., 2016). In the current investigation, both lethal and non-lethal chromosome mutations were observed. This is in concordance with earlier reports by Nair and Mehta (2014); Gnanamurthy and Dhanavel (2014), and Sahrish et al. (2019) in cowpea treated with EMS. Similar results were obtained in the studies conducted by Arisha et al. (2015) when pepper was subjected to EMS treatment and Thilagavathi and Mullainathan (2009) in black gram. Previous studies reported that chlorophyll development seems to be wild type led by many genes that are located on different chromosomes (Larkin and Scowcroft, 1981; Wang et al., 2013). EMS may have caused mutations in these genes. Mutations affecting the production of chlorophyll are important for identifying gene function and the clarification of chlorophyll metabolism and its regulation (Wu et al., 2007).

### Putative mutants of quantitative traits

Quantitative traits vary in degree and can be attributed to polygenic effects (product of two or more genes), and their environment. Hence, individuals with varying forms of these traits can be regarded as putative mutants (not necessarily true mutants) (Forster and Shu, 2011).

The application of a mutagen may cause genetic variations through breaking of linkage present in the genetic material resulting in the production of useful traits in crop species (Shah et al., 2008). Induction of mutation with EMS has resulted in the acquisition of important morpho-agronomic traits in cowpea production. Selection of putative mutants for quantitative traits is an important step for mutant development. Early mutants with 38–41 DTF were isolated in the M_2_ population in the present investigation. Early maturing mutants are very important. This is because they are able to escape drought or tolerate insect or pest damage because of their short duration of reproductive phase. Previous studies by Gnanamurthy and Dhanavel (2014) and Nair and Mehta (2014) isolated early maturing mutants from cowpea mutagenized with EMS. This indicates that EMS caused mutation in the gene responsible for this trait.

The current research revealed plants with high yield and low yield performance in the M_2_ population. Highly significant mutants that showed high performance for number of pods per plant, number of seeds per pod per plant, number of seeds per plant, number of locules per pod per plant and pod length were obtained in the M_2_ population. Top twenty mutants with high yield performance in number of seeds per plant were selected. Traits including higher number of pods per plant, increased protein content, plant height and high seed weights have been obtained in earlier works by Odeigah et al. (1998) and Singh et al. (2006) in cowpea mutagenesis with EMS. High number of pods per plant was reported by Gnanamurthy and Dhanavel (2014) in cowpea. The high yield performance may be as result of EMS altering the gene(s) for this trait. The higher performance in other yield traits exhibited by mutants may be as a result of pleiotropy and mutation in one trait affected the other.

There were highly significant mutants that showed lower performance for number of pods per plant, number of seeds per pod per plant, number of seeds per plant, number of locules per pod per plant and pod length compared to the wildtype. Reduction of number of yield characters were observed in the earlier studies by Jagajanantham et al. (2013) in *Abelmoschus esculentus* L. Moench. Previous studies by Berenschot et al. (2009) reported a reduction in number of seeds per pod and plant in *Petunia hybrid.* According to Alan (2007), induced mutations play prominent role in altering the genetic make-up of genotypes not only at a chromosomal but even at a molecular level. This alteration may improve on economically important trait. However, frequency of desirable mutations was very low, about 0.1 per cent of the total mutations. Mutations in quantitative traits are normally in the direction away from the selection history of the parent variety, example yield.

## Conclusion

In the current study, EMS was used to induce genetic variability in the “*Asontem*” cultivar of *Vigna unguiculata*. The EMS induced mutations were observed in the cultivar as significant differences when putative mutants are compared with the wildtype. Based on germination data, the lethal dose 50% was estimated to be at 0.4% concentration of EMS solution. There was decrease in percentage germination and survival due to the EMS mutagenesis. During the M_1_ generation there were differences between the mutant and the wild type populations. Visible mutations of the morphological traits were observed in the M_1_ populations. There were differences in growth habit, plant pigmentation, pod curvature, seed shape and seed coat color. Distribution of quantitative traits studied showed wide ranges in the mutant population as compared with the wild type. High frequency was observed for farmer preferred traits: erect growth habit and low frequency was observed for consumer preferred trait: cream seed coat color. During the M_2_ generation, the variations in the mutant populations continued. There was decrease in percentage germination and survival. A chlorophyll defect was observed at the seedling stage; albino, xantha and pale green plants were observed in the M_2_ population. There were visible mutations observed in leaf architecture in which some plants had different leaflet arrangement and number and irregular leaves structure. There were different levels of variation among the cowpea morphological traits observed. There were variations in leaf color, leaf shape, plant pigmentation, growth habit, twinning tendency, pod curvature, seed shape and seed coat color. There was high frequency for erect growth habit in the mutant population as compared with the wild type. There were wider distributions of quantitative traits in the mutant population. In order to genetically improve “*Asontem”* cultivar of cowpea, molecular work is required to analyse mutants that showed distinctive features to determine the genetic reasons underlying the changes observed. The EMS mutagenesis was effective in inducing the variations that will be useful for breeding and development of new farmer preferred varieties.

## Data availability statement

The original contributions presented in the study are included in the article/Supplementary material, further inquiries can be directed to the corresponding author.

## Author contributions

MOG and JE designed the project. MOG, LS, and FO-A performed the experiments. MOG and IA analyzed the data. MOG drafted the manuscript. JE and ED revised the manuscript. All authors contributed to the article and approved the submitted version.

## References

[B1] Alcantara T. Bosland P. Smith D. (1996). Ethyl methanesulfonate-induced seed mutagenesis of *Capsicum annuum*. *J. Heredity* 87 239–241. 10.1093/oxfordjournals.jhered.a022992

[B2] Adamu A. K. Aliyu H. (2007). Morphological effects of sodium azide on tomato (*Lycopersicon esculentum* Mill). *Sci. World J.* 2 9–12.

[B3] Alan H. S. (2007). Molecular markers to assess genetic diversity. *Euphytica* 158 313–321.

[B4] Ariramana M. Gnanamurthy S. Dhanavelb D. Bharathi T. Murugan S. (2014). Mutagenic effect on seed germination, seedling growth and seedling survival of Pigeon pea (*Cajanus cajan* (L.) Millsp). *Int. Lett. Nat. Sci.* 16 41–49. 10.18052/www.scipress.com/ILNS.21.41

[B5] Arisha M. H. Liang B.-K. Shah S. N. M. Gong Z.-H. Li D.-W. (2014). Kill curve analysis and response of first generation *Capsicum annuum* L. B12 cultivar to ethyl methane sulfonate. *Genet. Mol. Res.* 13 10049–10061. 10.4238/2014.November.28.9 25501216

[B6] Arisha M. H. Shah S. N. M. Gong Z. Jing H. Li C. Zhang H. (2015). Ethyl methane sulfonate induced mutations in M2 generation and physiological variations in M1 generation of peppers (*Capsicum annuum* L.). *Front. Plant Sci.* 6:399. 10.3389/fpls.2015.00399 26089827 PMC4454883

[B7] Asante I. K. (1991). *Inheritance and Genetic linkage in the Cowpea (Vigna unguiculata (L) WALP).* M.Phil thesis. Accra: University of Ghana-Legon.

[B8] Auti S. G. Apparao B. J. (2009). “Induced mutagenesis in Mung bean (*Vigna radiata* (L.) Wilczek),” in *Induced Plant Mutations in the Genetic Era*, ed. Shu Q. Y. (Rome: Food and Agriculture Organization of the United Nations), 97–100.

[B9] Baghery M. A. Kazemitabar S. K. Kenari R. E. (2016). Effect of EMS on germination and survival of okra (*Abelmoschus esculentus* L.). *Biharean Biol.* 10 33–36.

[B10] Berenschot A. Zucchi M. Tulmann-Neto A. Quecini V. (2009). Mutagenesis in *Petunia hybrida* Vilm. and isolation of a novel morphological mutant. *Brazil. J. Plant Physiol.* 20 95–103. 10.1590/S1677-04202008000200002

[B11] Bhat T. A. Khan A. H. Parveen S. (2005). Comparative analysis of meiotic abnormalities induced by gamma rays, EMS and MMS in *Vicia faba* L. *J. Ind. Bot. Soc.* 84 45–48.

[B12] Bind D. Dwivedi V. K. (2014). Effects mutagenesis on germination, plant survival and pollen sterility in M1 generation of cowpea (*Vigna unguiculata* L. Walp). *Indian J. Agric. Res.* 48 398–401. 10.5958/0976-058X.2014.01322.5

[B13] Danso K. E. Safo-Katanka O. Adu-Ampomah Y. Oduro V. Amoatey H. M. Asare O. K. (2008). “Application of induced mutation techniques in Ghana: impact, challenges and the future,” in *Proceedings of the FAO/IAEA International Symposium on Induced Mutations in Plants*, Vienna.

[B14] Devmani B. Dwivedi V. K. Singh S. K. (2016). Induction of chlorophyll mutations through physical and chemical mutagenesis in cowpea [*Vigna unguiculata* (L.) Walp.]. *Int. J. Adv. Res.* 4 49–53.

[B15] Dhanavel D. Girija M. (2009). Effect of EMS, DES and SA on quantitative traits of cowpea (*Vigna unguiculata* (L.) Walp.) in M1 generation. *Crop Res.* 37 239–241.0.

[B16] Dhanavel D. Pavadai P. Mullainathan L. Mohana D. Raju G. Girija M. (2008). Effectiveness and efficiency of chemical mutagens in Cowpea [*Vigna unguiculata* (L.) Walp.]. *Afr. J. Biotech.* 7 4116–4117.

[B17] Egbadzor K. F. Ofori K. Yeboah M. Aboagye L. M. Opoku-Agyeman M. O. Danquah E. Y. (2017). Diversity in 113 Cowpea [*Vigna unguiculata* (L) Walp] accessions assessed with 458 SNP markers. *Springer Plus* 3:541. 10.1/86/2193-180-3-541 PMC4190189 25332852

[B18] Forster B. P. Shu Q. Y. (2011). “Plant mutagenesis in crop improvement: basic terms and applications,” in *Plant Mutation Breeding and Biotechnology*, eds Shu Q. Y. Forster B. P. Nakagawa H. (Wallingford: CABI), 9–20. 10.1079/9781780640853.0009

[B19] Gaur S. Singh M. Rathore N. Bhati P. S. Kumar D. (2003). “Radiobiological responses of cowpea,” in *Advances in Arid Legumes Research*, ed. Henry A. (Jodhpur: Indian Arid Legumes Society, Central Arid Zone Research Institute), 75–78.

[B20] Gnanamurthy S. Dhanavel D. (2014). Effect of EMS on induced morphological mutants and chromosomal variation in Cowpea (*Vigna unguiculata* L.). *Int. Lett. Nat. Sci* 22 33–43. 10.18052/www.scipress.com/ILNS.22.33

[B21] Gunckel J. E. Sparrow A. H. (1961). “Ionizing radiation: biochemical, physiological and morphological aspects of their effects on plants,” in *Encycl. Plant Physiol*, ed. Ruhland W. (Berlin: Springer-Verlag), 555–611.

[B22] Hohmann U. Jacobs G. Jung C. (2005). An EMS mutagenesis protocol for sugar beet and isolation of non-bolting mutants. *Plant Breed.* 124 317–321. 10.1111/j.1439-0523.2005.01126.x

[B23] IAEA (2021). *Fact Sheets.* Vienna: IAEA.

[B24] Jagajanantham N. Dhanavel D. Gnanamurthy S. Pavadai P. (2013). Induced on chemical mutagens in Bhendi, *Abelmoschus esculentus* L. moench. *Int. J. Curr. Sci.* 5 133–137.

[B25] Khan H. Viswanatha K. P. Sowmya H. C. (2015). Study of genetic variability parameters in cowpea [*Vigna unguiculata* (L.) Walp.] germplasm lines. *Int. Q. J. Life Sci.* 10 747–750.

[B26] Kumar V. A. Kumari R. U. Amutha R. Kumar T. S. Hepziba S. J. Kumar C. R. A. (2009). Effect of chemical mutagen on expression of characters in arid legume pulse-68 cowpea (*Vigna unguiculata* L. Walp.). *Res. J. Agric. Biol. Sci.* 5 1115–1120.

[B27] Lackyan T. S. Dalvi V. V. (2015). Inheritance study of qualitative and quantitative traits in cowpea (*Vigna unguiculata* (L.). Walp.). *Int. J. Sci. Res.* 4 2170–2173.

[B28] Larkin P. J. Scowcroft W. (1981). Somaclonal variation a novel source of variability from cell cultures for plant improvement. *Theor. Appl. Genet.* 60 197–214. 10.1007/BF02342540 24276737

[B29] Maguire J. D. (1962). Speed of germination-aid in selection and evaluation for seedling emergence and vigour. *Crop Sci.* 2 176–177. 10.2135/cropsci1962.0011183X000200020033x

[B30] Matos Filho C. H. A. Gomes R. L. F. Freire Filho F. R. Rocha M. M. Lopes A. C. A. Nunes J. A. R. (2014). Inheritance of traits related to plant architecture in cowpea. *Cienc. Rural* 44 599–604. 10.1590/S0103-84782014000400004

[B31] Miller P. D. Vaughn K. C. Wilson K. G. (1984). Ethyl methane sulfonate-induced chloroplast mutagenesis in crops. *J. Heredity* 75 86–92. 10.1093/oxfordjournals.jhered.a109900

[B32] Moh C. C. (1971). Mutation breeding in seed-coat colors of beans (*Phaseolus vulgaris* L.). *Euphytica* 20 119–125. 10.1007/BF00146782

[B33] Muller H. J. (1927). Artificial transmutation of the gene. *Science* 66 84–87. 10.1126/science.66.1699.84 17802387

[B34] Nair R. Mehta A. K. (2014). Induced mutagenesis in Cowpea (*Vigna unguiculata* L. Walp) var. Arka Garima. *Indian J. Agric. Res.* 48 247–257. 10.5958/0976-058X.2014.00658.1

[B35] Nawale S. R. Apte U. B. Jadhav B. B. (2006). Effect of gamma-rays and ethyl methane sulfonate on seed germination and survival of seedlings in cowpea (*Vigna unguiculata* (L.) Walp). *J. Arid Legumes* 3 102–105.

[B36] Odeigah P. G. C. Osanyinpeju A. O. Myers G. O. (1998). Induced mutations in cowpea *Vigna unguiculata* (Leguminosae). *Rev. Biol. Trop.* 46 579–586.

[B37] Padi F. K. (2003). Genetic analysis of pigmentation in cowpea. *Pak. J. Biol.* 6 1655–1659. 10.3923/pjbs.2003.1655.1659

[B38] Page D. R. Grossniklaus U. (2002). The art and design of genetic screens: *Arabidopsis thaliana*. *Nat. Rev. Genet.* 3 124–136. 10.1038/nrg730 11836506

[B39] Predieri S. (2001). Mutation induction and tissue culture in improving fruits. *Plant Cell Tissue Organ Cult.* 64 185–210.

[B40] Ramya B. Nallathambi G. Ganesh Ram S. (2014). The effect of mutagens on M1 population of black gram (*Vigna mungo* L. Hepper). *Afr. J. Biotechnol.* 13 951–956.

[B41] Redei G. P. Acedo G. N. Sandhu S. S. (1984). “Mutation induction and detection in *Arabidopsis*,” in *Mutation Cancer, and Malformation*, eds Ehy C. Generoso W. M. (New York, NY: Plenum), 285–313. 10.1007/978-1-4613-2399-0_15

[B42] Ribeiro H. L. C. Boiteux L. S. Santos C. A. F. (2014). Genetic parameters of earliness and plant architecture traits suitable for mechanical harvesting of cowpea (*Vigna unguiculata*). *Austral. J. Crop Sci.* 8 1232–1238.

[B43] Roychowdhury R. Tah J. (2013). “Mutagenesis-a potential approach for crop improvement,” in *Crop Improvement: New Approaches and Modern Techniques*, 1st Edn, eds Hakeem K. R. Ahmad P. Ozturk M. (New York, NY: Springer Science Business Media), 149–187. 10.1007/978-1-4614-7028-1_4

[B44] Sahrish F. Neha T. Choudhary S. Narayan P. (2019). Mutation breeding in cowpea *Vigna unguiculata* (L.) Walp. (Fabaceae). *Int. J. Univ. Sci. Technol.* 05:47.

[B45] Serrat X. Esteban R. Guibourt N. Moysset L. Nogués S. Lalanne E. (2014). EMS mutagenesis in mature seed-derived rice calli as a new method for rapidly obtaining TILLING mutant populations. *Plant Methods* 10:5. 10.1186/1746-4811-10-5 24475756 PMC3923009

[B46] Shah T. Mirza J. Haq M. Atta B. (2008). Radio sensitivity of various chickpea genotypes in M1 generation I-laboratory studies. *Pak. J. Bot.* 40 649–665.

[B47] Shu Q. Forster B. Nakagawa H. (2012). *Plant Mutation Breeding and Biotechnology.* Wallingford: CABI. 10.1079/9781780640853.0000

[B48] Sikora P. Chawade A. Larsson M. Olsson J. Olsson O. (2011). Mutagenesis as a tool in plant genetics, functional genomics, and breeding. *Int. J. Plant Genom.* 2011:314829. 10.1155/2011/314829 22315587 PMC3270407

[B49] Singh V. V. Ramkrishna K. Kumar Arya R. (2006). Induced chemical mutagenesis in cowpea (*Vigna unguiculata* L. Walp). *Indian J. Genet.* 66 312–315.

[B50] Stadler L. J. (1928a). Mutations in barley induced by x-rays and radium. *Science* 68 186–197. 10.1126/science.68.1756.186 17774921

[B51] Stadler L. J. (1928b). Genetic effects of X-rays in maize. *Proc. Natl. Acad. Sci. U.S.A.* 14 69–75. 10.1073/pnas.14.1.69 16587308 PMC1085350

[B52] StataCorp (2015). *Stata Statistical Software: Release 14.* College Station, TX: StataCorp LP.

[B53] Thilagavathi C. Mullainathan L. (2009). Isolation of macro mutants and mutagenic effectiveness, efficiency in Black gram (*Vigna mungo* (L.) Hepper). *Glob. J. Mol. Sci.* 4 76–79.

[B54] Ugorji O. U. Ikpeme E. V. Obu J. A. Ekpenyong E. D. (2012). Assessing the mutagenic effects of gamma irradiation on *Cajanus cajan* (L.) Huth and *Vigna unguiculata* (L.) Walp landraces using morphological markers. *Comun. Sci.* 3 271–281.

[B55] Uma M. Salimath P. M. (2001). Effect of ionizing radiation on germination and emergence of cowpea seeds. *Karnataka J. Agric. Sci.* 14 1063–1064.

[B56] Wang L. N. Zhang B. Li J. Yang X. Ren Z. (2014). Ethyl methane sulfonate (EMS) mutagenesis of cucumber (*Cucumis sativus* L.). *J. Agric. Sci.* 5 716–721.

[B57] Wang Z.-K. Huang Y.-X. Miao Z.-D. Hu Z.-Y. Song X.-Z. Liu L. (2013). IdentificationandcharacterizationofBGL11(t), a novel gene regulating leaf- colour mutation in rice (*Oryza sativa* L.). *Genes Genom.* 35 491–499. 10.3923/ajps.2009.318.321

[B58] Wani A. (2009). Mutagenic effectiveness and efficiency of gamma rays, Ethyl Methane Sulphonate and their combination treatments in Chickpea (*Cicer arietinum* L.). *Asian J. Plant Sci.* 8 318–321. 10.3923/ajps.2009.318.321

[B59] Wani A. A. Anis M. (2008). Gamma ray- and EMS-induced bold-seeded high-yielding mutants in chickpea (*Cicer arietinum* L.). *Turk. J. Biol.* 32 161–166.

[B60] Wu Z. Zhang X. He B. Diao L. Sheng S. Wang J. (2007). A chlorophyll- deficient rice mutant with impaired chlorophyllide esterification in chlorophyll biosynthesis. *Plant Physiol.* 145 29–40. 10.1104/pp.107.100321 17535821 PMC1976586

