# Supplementary material for: Induced variations of ethyl methane sulfonate mutagenized cowpea (Vigna unguiculata L. walp) plants
Source: Front Plant Sci. 2022 Aug 5;13:952247. doi: 10.3389/fpls.2022.952247 (PMC9394701; doi:10.3389/fpls.2022.952247)
Supplement: Supplementary file 1 [file Data_Sheet_1.docx]

Supplementary Material

**APPENDICES**

**Appendix 1:** **Protocol for Ethyl Methane Sulfonate (EMS) Mutagenesis of Cowpea Seeds**

Materials

1. Seeds or germ containing plant part
2. Ethyl methane sulfonate, EMS
3. Sodium thiosulfate
4. Distilled water

A. Prepare up to 5 liters of 1M Sodium thiosulphate

1. In 1 liter add 248.18g of sodium thiosulfate and then heat and stir until dissolution.
2. Repeat for 3 times
3. Move rotating vortex into a cleaned fume hood
4. Get googles, nose mask, and protection clothing ready for everyone
5. Move 1000µl pipette and tips into fume hood.

B. Prepare 0.4% of EMS solution into 50ml of H_2_0

1. Place 50ml bottle under the hood and
2. Add exactly 49.80ml of H_2_0
3. Add the cowpea seeds (about 100 seeds = 50ml)
4. Add 200 µl of EMS solution
5. Close bottle tightly and mix thoroughly
6. Put on rotating vertical vortex at 200rpm for 12 – 16 hours
7. Clean hood with Sodium thiosulfate

C. Terminating the experiment

1. Add 100ml sodium thiosulfate solution to neutralize and stop the reaction by inactivating the EMS
2. Keep for 5 minutes on the shaker and dispense the solution into a well labelled restricted container.
3. Add 100ml of H_2_O to make the seeds sink
4. Wash 3 times with 100ml H_2_o for 5-7 minutes each by gently turning the tube upside down for a couple of times.
5. Clean everything with 1M sodium thiosulfate and rinse with water.

*Note liquid waste should be restricted to and contained in a clearly labeled bottle with inscriptions “EMS treated water” for disposal.

E. Intermediate sowing

Sow in a well-watered soil.

Preparation of EMS concentrations, Total volume = 50ml

1. For 0.2%, add 100 µl of EMS solution in a total volume 50 ml (H_2_O=49.9ml volume)
2. For 0.4%, add 200 µl of EMS solution in a total volume 50 ml (H_2_O=49.8ml volume)
3. For 0.6%, add 300 µl of EMS solution in a total volume 50 ml (H_2_O=49.7ml volume)
4. For 0.8%, add 400 µl of EMS solution in a total volume 50 ml (H_2_O=49.6ml volume)

**Appendix 2 Morphological Characterization of M_1_ and M_2_ Populations**

**Qualitative data**

**Leaf Colour:** The intensity of green colour of the leaflet for each plant was observed and recorded using colour chart.

**Leaf markings**: Leaves with ‘v’ markings were observed and recorded at the sixth weeks after planting.

**Leaf shape**: The leaflet shape for each plant was obtained and recorded (IBPGR, 1983).

**Plant pigmentation:** The pigmentation of the petiole, branch, stem, peduncle for each plant was determined.

**Flower colour**: The colour of the flowers of the individual plants was observed and recorded.

**Growth habit:** The growth habits for each plant within the wild type and treatments were determined.

**Growth pattern:** With the help of the descriptor, plants with different growth pattern were observed and recorded

**Twinning tendency:** The twinning tendency for each individual plant within the wild type and mutagenized population was observed and recorded.

**Seed coat colour:** Seed coat colour of individual plants was determined and recorded.

**Seed shape:** The shapes of seeds from individual plants within each treatment were determined.

**Pod colour:** Colour of the matured pod was observed and recorded using the cowpea descriptor and colour chart.

**Quantitative data**

**Chlorophyll content of leaves**: Chlorophyll content was measured using the chlorophyll meter. The median leaflet of the trifoliate was measured and an average was taken to record the chlorophyll content for each of the individual plants within the wild type and treatments.

**Leaf mutant**: Plants with different compound leave aside the trifoliate leaves were observed within the wild type and treatments. The number of leaflets (monofoliate, bifoliate, tetrafoliate, pentofoliate etc.) for the mutant leaves was recorded.

**Days to flowering**: This was observed by counting the number of days it took an individual to form the first flower after planting.

**Plant height**: The plant height measurement was taken as the distance between the first node and apex of the plant. This measurement was done by using measuring tape.

**Pod Length:** Three (3) pods of each plant in the wild type and mutant population were measured using a string from the stylar end to the point of attachment of the pod to the stalk. The string was stretched on a 30 cm rule and the pod length was read. The mean pod length calculated and recorded.

**Number of seed per pod**: Three pods were opened by the hand and the number of seeds in each pod per individual plants within wild type and mutant population was counted. Total number of seeds was recorded and the mean was calculated.

**Number of pods per plant:** Total number of pods from each plant was counted after harvesting. Harvesting was done once when 80% of the pods were matured.

**Number of seeds per plant**: this was estimated in excel by multiplying number of pods per plant by average number of seeds per pods per individual plant.

**Percentage germination:** The number of seeds germinated among the wild type and treatments were observed. The percentage seed germination was calculated and recorded.

**Frequency distribution of qualitative traits in M_1_ generation**

|  |  | **frequency (%)** | |
| --- | --- | --- | --- |
| **Trait** | **phenotypic class** | **Wild type** | **M_1_** |
| **Stem pigmentation** | None | 73.03 | 78.91 |
|  | Very slight | 4.61 | 5.45 |
|  | Moderate | 6.58 | 3.79 |
|  | Intermediate | 7.24 | 5.69 |
|  | Extensive | 8.55 | 3.55 |
|  | Solid | 0.00 | 2.61 |
| **Branch pigmentation** | None | 15.13 | 28.91 |
|  | Very slight | 5.26 | 4.98 |
|  | Moderate | 39.47 | 30.09 |
|  | Intermediate | 29.61 | 24.41 |
|  | Extensive | 9.87 | 9.95 |
|  | Solid | 0.66 | 1.66 |
| **Petiole pigmentation** | None | 77.63 | 84.60 |
|  | Very slight | 12.50 | 10.90 |
|  | Moderate | 6.58 | 4.50 |
|  | Intermediate | 3.29 | 0.00 |
|  | Extensive | 0.00 | 0.00 |
|  | Solid | 0.00 | 0.00 |
| **Leaf shape** | Hastate | 100 | 100.00 |
| **Growth habit** | Acute erect | 0.00 | 7.82 |
|  | Erect | 0.00 | 18.96 |
|  | Semi-erect | 0.00 | 2.13 |
|  | Intermediate | 100 | 12.09 |
|  | Semi-prostrate | 0.00 | 12.80 |
|  | Prostrate | 0.00 | 14.45 |
|  | Climbing | 0.00 | 31.75 |
| **Flower colour** | Violet | 100.00 | 100 |
| **Pod curvature** | Straight | 0.00 | 37.20 |
|  | Slightly curved | 100.00 | 37.68 |
|  | Curved | 0.00 | 25.12 |
| **Mature pod colour** | Pale tan | 100 | 100 |
| **Seed shape** | Rhombus | 100.00 | 22.17 |
|  | Kidney | 0.00 | 0.24 |
|  | Ovoid | 0.00 | 77.59 |
| **Seed coat colour** | Walnut | 100 | 72.05 |
|  | chocolate | 0 | 27.47 |
|  | Cream | 0 | 0.48 |

**Frequency distribution of qualitative Traits in the M_2_ Population**

|  |  | **Frequency(%)** | |
| --- | --- | --- | --- |
| **Trait** | **Phenotypic class** | **Wild type** | **M2** |
| Leaf colour | Pale green | 0.00 | 1.13 |
|  | Intermediate green | 0.00 | 20.74 |
|  | Dark green | 100.00 | 78.12 |
| Terminal leaf shape | Hastate | 100.00 | 92.88 |
|  | Sub-hastate | 0.00 | 7.12 |
| Leaf marking | Absent | 0.00 | 0.00 |
|  | Present | 100.00 | 100.00 |
| Plant pigmentation | None | 0.00 | 2.55 |
|  | Very slight | 18.75 | 40.41 |
|  | Moderate | 61.25 | 39.51 |
|  | Intermediate | 17.50 | 13.11 |
|  | Extensive | 2.50 | 4.1 |
|  | Solid | 0.00 | 0.33 |
| Growth pattern | Determinate | 0.00 | 0.00 |
|  | Indeterminate | 100.00 | 100.00 |
| Flower colour | Violet | 100.00 | 100.00 |
|  | White | 0.00 | 0.00 |
| Growth habit | Acute erect | 0.00 | 32.91 |
|  | Erect | 0.00 | 9.34 |
|  | Semi-erect | 0.00 | 7.45 |
|  | Intermediate | 100.00 | 6.08 |
|  | Semi-prostrate | 0.00 | 38.33 |
|  | Prostrate | 0.00 | 3.21 |
|  | Climbing | 0.00 | 2.69 |
| Twinning tendency | None | 0.00 | 53.89 |
|  | Slight | 17.50 | 28.29 |
|  | Intermediate | 82.50 | 15.13 |
|  | Pronounced | 0.00 | 2.69 |
| Pod curvature | Straight | 0.00 | 65.39 |
|  | Slightly curved | 100.00 | 33.99 |
|  | Curved | 0.00 | 0.61 |
| Pod colour | Pale tan/Straw | 100.00 | 100.00 |
| Seed shape | Kidney | 0.00 | 2.03 |
|  | Ovoid | 0.00 | 47.67 |
|  | Crowder | 0.00 | 0 |
|  | Globose | 0.00 | 0.05 |
|  | Rhomboid | 100.00 | 50.07 |
| Seed coat colour | walnut | 100.00 | 85.48 |
|  | chocolate | 0.00 | 14.43 |
|  | coffee | 0.00 | 0.09 |

**Appendix 3: Summary Statistics of Quantitative Traits in the M_1_ Mutagenized Population.**

CC=Chlorophyll content, PH= Plant height, NOB=Number of branches, DTF= Days to flowering, DTFMP=Days to first maturing pod, NOPP= number of pods per plant, PL= Pod length, NPPP=number of peduncles per plant, SW=seed weight, NOLP=Number of Locules per plant.

|  | **CC** | **PH** | **DTF** | **NOPP** | **PL** | **NPPP** | **NOSP** | **NOLP** |
| --- | --- | --- | --- | --- | --- | --- | --- | --- |
| **Minimum** | 10.4 | 20.0 | 60 | 1 | 3.0 | 1 | 1 | 6 |
| **1st Quimum** | 31.9 | 82.25 | 61 | 6 | 13.6 | 1 | 5 | 10 |
| **Median** | 36.45 | 113.0 | 62 | 12 | 14.3 | 1 | 9 | 11 |
| **Mean** | 36.86 | 110.38 | 62 | 18 | 14.2 | 2 | 8 | 11 |
| **3rd Quimum** | 41.9 | 139.0 | 62 | 25 | 15.0 | 2 | 11 | 12 |
| **Maximum** | 56.9 | 200.0 | 66 | 80 | 18.2 | 3 | 14 | 20 |
| **NA's** | 0 | 0.0 | 0 | 59 | 59 | 59 | 59 | 59 |
| **Total Observed** | 478 | 478.0 | 478 | 419 | 419 | 419 | 419 | 419 |

**Appendix 4: Summary Statistics of Quantitative Traits in the Wild type (M_1_ Generation).**

CC=Chlorophyll content, PH= Plant height, NOB=Number of branches, DTF= Days to flowering, DTFMP=Days to first maturing pod, NOPP= number of pods per plant, PL= Pod length, NPPP=number of peduncles per plant, SW=seed weight, NOLP=Number of Locules per plant.

|  | **CC** | **PH** | **DTF** | **NOPP** | **PL** | **NPPP** | **NOSP** | **NOLP** |
| --- | --- | --- | --- | --- | --- | --- | --- | --- |
| **Minimum** | 20.90 | 100.0 | 59 | 18 | 12.9 | 1 | 7 | 10 |
| **1st Quimum** | 33.15 | 136.9 | 62 | 37 | 14.4 | 1 | 10 | 12 |
| **Median** | 35.70 | 160.5 | 62 | 40 | 15.4 | 2 | 11 | 12 |
| **Mean** | 37.04 | 159.7 | 62 | 39 | 15.4 | 2 | 11 | 12 |
| **3rd Quimum** | 41.77 | 184.2 | 62 | 43 | 16.3 | 2 | 12 | 13 |
| **Maximum** | 51.00 | 200.0 | 65 | 46 | 18.1 | 2 | 13 | 14 |
| **Total Observed** | 30 | 30 | 30 | 30 | 30 | 30 | 30 | 30 |

**Appendix 5: Germination count and Percentage Germination of Mutant Lines and Wild type.**

| **Lines** | **Germination count** | **Percentage Germination** | **Germination speed** | |
| --- | --- | --- | --- | --- |
| A1 | 16 | 80 | 1.6 |  |
| A2 | 16 | 80 | 1.6 |  |
| A3 | 15 | 75 | 1.5 |  |
| A4 | 15 | 75 | 1.5 |  |
| A5 | 15 | 75 | 1.5 |  |
| A6 | 16 | 80 | 1.6 |  |
| A7 | 16 | 80 | 1.6 |  |
| A8 | 18 | 90 | 1.8 |  |
| A9 | 20 | 100 | 2 |  |
| A10 | 17 | 85 | 1.7 |  |
| A11 | 17 | 85 | 1.7 |  |
| A12 | 20 | 100 | 2 |  |
| A13 | 17 | 85 | 1.7 |  |
| A14 | 3 | 15 | 0.3 |  |
| A15 | 18 | 90 | 1.8 |  |
| A16 | 19 | 95 | 1.9 |  |
| A17 | 19 | 95 | 1.8 |  |
| A18 | 18 | 90 | 1.8 |  |
| A19 | 19 | 95 | 1.9 |  |
| A20 | 11 | 55 | 1.1 |  |
| A21 | 16 | 80 | 1.6 |  |
| A22 | 19 | 95 | 1.9 |  |
| A23 | 6 | 30 | 0.6 |  |
| A24 | 19 | 95 | 1.9 |  |
| A25 | 17 | 85 | 1.7 |  |
| A26 | 19 | 95 | 1.9 |  |
| A27 | 18 | 90 | 1.8 |  |
| A28 | 20 | 100 | 2 |  |
| A29 | 19 | 95 | 1.9 |  |
| A30 | 15 | 75 | 1.5 |  |
| A31 | 16 | 80 | 1.6 |  |
| A32 | 18 | 90 | 1.8 |  |
| A33 | 12 | 60 | 1.2 |  |
| A34 | 18 | 90 | 1.8 |  |
| A35 | 12 | 60 | 1.2 |  |
| A36 | 17 | 85 | 1.7 |  |
| A37 | 15 | 75 | 1.5 |  |
| A38 | 14 | 70 | 1.4 |  |
| A39 | 17 | 85 | 1.7 |  |
| A40 | 18 | 90 | 1.8 |  |
| A41 | 17 | 85 | 1.7 |  |
| A42 | 14 | 70 | 1.4 |  |
| A43 | 17 | 85 | 1.7 |  |
| A44 | 18 | 90 | 1.8 |  |

Appendix 5 (cont’d)

| **Lines** | **Germination count** | **Percentage Germination** | **Germination speed** | |
| --- | --- | --- | --- | --- |
| A45 | 14 | 70 | 1.4 |  |
| A46 | 12 | 60 | 1.2 |  |
| A47 | 19 | 95 | 1.9 |  |
| A48 | 19 | 95 | 1.9 |  |
| A49 | 9 | 45 | 0.9 |  |
| A50 | 17 | 85 | 1.7 |  |
| A51 | 19 | 95 | 1.9 |  |
| A52 | 15 | 75 | 1.5 |  |
| A53 | 18 | 90 | 1.8 |  |
| A54 | 16 | 80 | 1.3 |  |
| A55 | 9 | 45 | 0.9 |  |
| A56 | 11 | 55 | 1.1 |  |
| A57 | 10 | 50 | 0.9 |  |
| A58 | 6 | 30 | 0.5 |  |
| A59 | 14 | 70 | 1.4 |  |
| A60 | 15 | 75 | 1.5 |  |
| A61 | 16 | 80 | 1.5 |  |
| A62 | 12 | 60 | 1.2 |  |
| A63 | 16 | 80 | 1.3 |  |
| A64 | 15 | 75 | 1.4 |  |
| B1 | 13 | 65 | 1.3 |  |
| B2 | 15 | 75 | 1.4 |  |
| B3 | 13 | 65 | 1.2 |  |
| B4 | 14 | 70 | 1.4 |  |
| B5 | 16 | 80 | 1.6 |  |
| B6 | 17 | 85 | 1.7 |  |
| B7 | 17 | 85 | 1.7 |  |
| B8 | 13 | 65 | 1.2 |  |
| B9 | 14 | 70 | 1.3 |  |
| B10 | 13 | 65 | 1.3 |  |
| B11 | 13 | 65 | 1.3 |  |
| B12 | 15 | 75 | 1.5 |  |
| B13 | 17 | 85 | 1.7 |  |
| B14 | 16 | 80 | 1.6 |  |
| B15 | 18 | 90 | 1.8 |  |
| B16 | 16 | 80 | 1.6 |  |
| B17 | 18 | 90 | 1.8 |  |
| B18 | 16 | 80 | 1.5 |  |
| B19 | 18 | 90 | 1.8 |  |
| B20 | 20 | 100 | 2 |  |
| B21 | 18 | 90 | 1.7 |  |
| B22 | 17 | 85 | 1.7 |  |
| B23 | 17 | 85 | 1.7 |  |
| B24 | 16 | 80 | 1.6 |  |
| B25 | 16 | 80 | 1.6 |  |

Appendix 5 (cont’d)

| **Lines** | **Germination count** | **Percentage Germination** | **Germination speed** | |
| --- | --- | --- | --- | --- |
| B26 | 13 | 65 | 1.3 |  |
| B27 | 14 | 70 | 1.4 |  |
| B28 | 10 | 50 | 1 |  |
| B29 | 18 | 90 | 1.8 |  |
| B30 | 19 | 95 | 1.9 |  |
| B31 | 16 | 80 | 1.6 |  |
| B32 | 16 | 80 | 1.6 |  |
| B33 | 12 | 60 | 1.2 |  |
| B34 | 17 | 85 | 1.7 |  |
| B35 | 10 | 50 | 0.9 |  |
| B36 | 14 | 70 | 1.4 |  |
| B37 | 17 | 85 | 1.7 |  |
| B38 | 12 | 60 | 1.2 |  |
| B39 | 15 | 75 | 1.5 |  |
| B40 | 12 | 60 | 1.2 |  |
| B41 | 19 | 95 | 1.8 |  |
| B42 | 18 | 90 | 1.8 |  |
| B43 | 9 | 45 | 0.9 |  |
| B44 | 15 | 75 | 1.5 |  |
| B45 | 16 | 80 | 1.5 |  |
| B46 | 18 | 90 | 1.7 |  |
| B47 | 11 | 55 | 0.9 |  |
| B48 | 14 | 70 | 1.3 |  |
| B49 | 19 | 95 | 1.7 |  |
| B50 | 18 | 90 | 1.8 |  |
| B51 | 18 | 90 | 1.8 |  |
| B52 | 14 | 70 | 1.2 |  |
| B53 | 16 | 80 | 1.6 |  |
| B54 | 17 | 85 | 1.7 |  |
| B55 | 12 | 60 | 1.2 |  |
| B56 | 19 | 95 | 0.9 |  |
| B57 | 17 | 85 | 1.6 |  |
| B58 | 13 | 65 | 1.3 |  |
| B59 | 15 | 75 | 1.5 |  |
| B60 | 11 | 55 | 1.1 |  |
| B61 | 10 | 50 | 1 |  |
| B62 | 13 | 65 | 1.3 |  |
| B63 | 13 | 65 | 1.2 |  |
| B64 | 12 | 60 | 1.2 |  |
| C1 | 17 | 85 | 1.7 |  |
| C2 | 16 | 80 | 1.6 |  |
| C3 | 18 | 90 | 1.7 |  |
| C4 | 16 | 80 | 1.4 |  |
| C5 | 14 | 70 | 1.3 |  |
| C6 | 15 | 75 | 1.5 |  |

Appendix 5 cont’d

| **Lines** | **Germination count** | **Percentage Germination** | **Germination speed** | |
| --- | --- | --- | --- | --- |
| C7 | 18 | 90 | 1.7 |  |
| C8 | 15 | 75 | 1.5 |  |
| C9 | 15 | 75 | 1.5 |  |
| C10 | 12 | 60 | 1.2 |  |
| C11 | 14 | 70 | 1.4 |  |
| C12 | 17 | 85 | 1.6 |  |
| C13 | 14 | 70 | 1.3 |  |
| C14 | 14 | 70 | 1.1 |  |
| C15 | 15 | 75 | 1.5 |  |
| C16 | 13 | 65 | 1.3 |  |
| C17 | 17 | 85 | 1.7 |  |
| C18 | 16 | 80 | 1.5 |  |
| C19 | 15 | 75 | 1.4 |  |
| C20 | 19 | 95 | 1.8 |  |
| C21 | 17 | 85 | 1.6 |  |
| C22 | 14 | 70 | 1.4 |  |
| C23 | 11 | 55 | 1.1 |  |
| C24 | 9 | 45 | 0.7 |  |
| C25 | 15 | 75 | 1.4 |  |
| C26 | 12 | 60 | 1.2 |  |
| C27 | 12 | 60 | 1.2 |  |
| C28 | 7 | 35 | 0.7 |  |
| C29 | 14 | 70 | 1.4 |  |
| C30 | 4 | 20 | 0.3 |  |
| C31 | 17 | 85 | 1.6 |  |
| C32 | 11 | 55 | 0.9 |  |
| C33 | 13 | 65 | 1.3 |  |
| C34 | 17 | 85 | 1.7 |  |
| C35 | 16 | 80 | 1.6 |  |
| C36 | 15 | 75 | 1.5 |  |
| C37 | 13 | 65 | 1.3 |  |
| C38 | 12 | 60 | 1.2 |  |
| C39 | 14 | 70 | 1.3 |  |
| C40 | 12 | 60 | 1.2 |  |
| C41 | 12 | 60 | 1.2 |  |
| C42 | 9 | 45 | 0.9 |  |
| C43 | 12 | 60 | 1.2 |  |
| C44 | 13 | 65 | 1.3 |  |
| C45 | 15 | 75 | 1.4 |  |
| C46 | 15 | 75 | 1.5 |  |
| C47 | 15 | 75 | 1.4 |  |
| C48 | 14 | 70 | 1.4 |  |
| C49 | 14 | 70 | 1.4 |  |
| C50 | 15 | 75 | 1.4 |  |
| C51 | 16 | 80 | 1.5 |  |

Appendix 5 (cont’d)

| **Lines** | **Germination count** | **Percentage Germination** | **Germination speed** | |
| --- | --- | --- | --- | --- |
| C52 | 16 | 80 | 1.6 |  |
| C53 | 13 | 65 | 1.3 |  |
| C54 | 12 | 60 | 1.3 |  |
| C55 | 11 | 55 | 1.1 |  |
| C56 | 12 | 60 | 1.1 |  |
| C57 | 12 | 60 | 1.2 |  |
| C58 | 15 | 75 | 1.5 |  |
| C59 | 14 | 70 | 1.4 |  |
| C60 | 14 | 70 | 1.4 |  |
| C61 | 15 | 75 | 1.4 |  |
| C62 | 14 | 70 | 1.4 |  |
| C63 | 14 | 70 | 1.3 |  |
| C64 | 10 | 50 | 0.9 |  |
| D1 | 17 | 85 | 1.6 |  |
| D2 | 20 | 100 | 2 |  |
| D3 | 18 | 90 | 1.7 |  |
| D4 | 11 | 55 | 1.1 |  |
| D5 | 17 | 85 | 1.6 |  |
| D6 | 14 | 70 | 1.4 |  |
| D7 | 11 | 55 | 1.1 |  |
| D8 | 15 | 75 | 1.4 |  |
| D9 | 16 | 80 | 1.4 |  |
| D10 | 15 | 75 | 1.4 |  |
| D11 | 17 | 85 | 1.6 |  |
| D12 | 13 | 65 | 1.3 |  |
| D13 | 13 | 65 | 1.3 |  |
| D14 | 17 | 85 | 1.6 |  |
| D15 | 16 | 80 | 1.4 |  |
| D16 | 15 | 75 | 1.4 |  |
| D17 | 17 | 85 | 1.6 |  |
| D18 | 16 | 80 | 1.5 |  |
| D19 | 11 | 55 | 1.1 |  |
| D20 | 19 | 95 | 1.9 |  |
| D21 | 17 | 85 | 1.7 |  |
| D22 | 19 | 95 | 1.9 |  |
| D23 | 16 | 80 | 1.5 |  |
| D24 | 19 | 95 | 1.9 |  |
| D25 | 16 | 80 | 1.5 |  |
| D26 | 17 | 85 | 1.7 |  |
| D27 | 13 | 65 | 1.2 |  |
| D28 | 16 | 80 | 1.6 |  |
| D29 | 18 | 90 | 1.7 |  |
| D30 | 15 | 75 | 1.4 |  |
| D31 | 16 | 80 | 1.6 |  |
| D32 | 19 | 95 | 1.9 |  |
| D33 | 13 | 65 | 1.2 |  |
| D34 | 14 | 70 | 1.5 |  |
| D35 | 16 | 80 | 1.6 |  |

Appendix 5 (cont’d)

| **Lines** | **Germination count** | **Percentage Germination** | **Germination speed** |
| --- | --- | --- | --- |
| D36 | 17 | 85 | 1.7 |
| D37 | 12 | 60 | 1.2 |
| D38 | 15 | 75 | 1.5 |
| D39 | 16 | 80 | 1.6 |
| D40 | 13 | 65 | 1.3 |
| D41 | 15 | 75 | 1.4 |
| D42 | 8 | 40 | 0.8 |
| D43 | 18 | 90 | 1.7 |
| D44 | 16 | 80 | 1.6 |
| D45 | 14 | 70 | 1.3 |
| D46 | 6 | 30 | 0.6 |
| D47 | 17 | 85 | 1.6 |
| D48 | 17 | 85 | 1.6 |
| D49 | 1 | 5 | 0.1 |
| D50 | 19 | 95 | 1.8 |
| D51 | 12 | 60 | 1.2 |
| D52 | 15 | 75 | 1.4 |
| D53 | 11 | 55 | 1 |
| D54 | 9 | 45 | 0.7 |
| D55 | 15 | 75 | 1.5 |
| D56 | 1 | 5 | 0.1 |
| D57 | 17 | 85 | 1.8 |
| D58 | 4 | 20 | 0.2 |
| D59 | 15 | 75 | 1.4 |
| D60 | 12 | 60 | 1 |
| D61 | 11 | 55 | 1.1 |
| D62 | 15 | 75 | 1.4 |
| D63 | 12 | 60 | 1.2 |
| D64 | 17 | 85 | 1.7 |
| E1 | 14 | 70 | 1.1 |
| E2 | 17 | 85 | 1.7 |
| E3 | 16 | 80 | 1.5 |
| E4 | 18 | 90 | 1.8 |
| E5 | 15 | 75 | 1.4 |
| E6 | 14 | 70 | 1.1 |
| E7 | 16 | 80 | 1.5 |
| E8 | 17 | 85 | 1.6 |
| E9 | 13 | 65 | 1.3 |
| E10 | 14 | 70 | 1.3 |
| E11 | 14 | 70 | 1.3 |
| E12 | 16 | 80 | 1.4 |
| E13 | 12 | 60 | 1 |
| E14 | 19 | 95 | 1.8 |
| E15 | 4 | 20 | 0.3 |
| E16 | 15 | 75 | 1.4 |
| E17 | 15 | 75 | 1.5 |
| E18 | 17 | 85 | 1.5 |
| E19 | 15 | 75 | 1.3 |
| E20 | 5 | 25 | 0.5 |
| E21 | 7 | 35 | 0.7 |
| E22 | 13 | 65 | 1.3 |
| E23 | 17 | 85 | 1.7 |

Appendix 5 cont’d

| **Lines** | **Germination count** | **Percentage Germination** | **Germination speed** |
| --- | --- | --- | --- |
| E25 | 14 | 70 | 1.4 |
| E26 | 11 | 55 | 1.1 |
| E27 | 12 | 60 | 1.1 |
| E28 | 18 | 90 | 1.7 |
| E29 | 19 | 95 | 1.8 |
| E30 | 15 | 75 | 1.5 |
| E31 | 18 | 90 | 1.8 |
| E32 | 13 | 65 | 1.3 |
| E33 | 20 | 100 | 2 |
| E34 | 17 | 85 | 1.7 |
| E35 | 18 | 90 | 1.8 |
| E36 | 20 | 100 | 2 |
| E37 | 16 | 80 | 1.6 |
| E38 | 13 | 65 | 1.3 |
| E39 | 17 | 85 | 1.7 |
| E40 | 19 | 95 | 1.7 |
| E41 | 14 | 70 | 1.4 |
| E42 | 17 | 85 | 1.7 |
| E43 | 18 | 90 | 1.8 |
| E44 | 18 | 90 | 1.8 |
| E45 | 13 | 65 | 1.2 |
| E46 | 15 | 75 | 1.5 |
| E47 | 16 | 80 | 1.6 |
| E48 | 17 | 85 | 1.5 |
| E49 | 17 | 85 | 1.6 |
| E50 | 16 | 80 | 1.5 |
| E51 | 18 | 90 | 1.8 |
| E52 | 18 | 90 | 1.6 |
| E53 | 17 | 85 | 1.7 |
| E54 | 12 | 60 | 1.2 |
| E55 | 11 | 55 | 1 |
| E56 | 14 | 70 | 1.4 |
| E57 | 20 | 100 | 2 |
| E58 | 16 | 80 | 1.6 |
| E59 | 11 | 55 | 1.1 |
| E60 | 12 | 60 | 0 |
| E61 | 10 | 50 | 1 |
| E62 | 13 | 65 | 0.8 |
| E63 | 13 | 65 | 1.3 |
| Wild type-1 | 15 | 75 | 1.5 |
| Wild type-2 | 18 | 90 | 1.8 |
| Wild type-3 | 15 | 75 | 1.5 |
| Wild type-4 | 13 | 65 | 1.3 |
| Wild type-5 | 17 | 85 | 1.7 |
| **Total** |  |  |  |
| **Mutagenized population** | 4723 | 74.03 | 1.43 |
| **Wild type** | 80 | 80 | 1.56 |

**Appendix 6: Summary Statistics of Quantitative Traits in the Wild type (M_2_ Generation).**

DTF= Days to flowering, NOPPP= number of pods per plant, NOSPP= Number of seeds per pod, PL= Pod length, NOLP=Number of Locules per plant, PSST=Percentage Seed Set, NSPP=Number of seeds per Plant

|  | **DTF** | **NOPPP** | **NOSPP** | **PL** | **NOLP** | **PSST** | **NSPP** |
| --- | --- | --- | --- | --- | --- | --- | --- |
| **Minimum** | 40 | 10 | 9 | 12.1 | 10 | 81.82 | 150 |
| **1st quartile** | 51 | 25 | 13 | 14.5 | 14 | 88.65 | 321 |
| **Median** | 54 | 28 | 14 | 15.7 | 15 | 93.33 | 377 |
| **Mean** | 54 | 28 | 12 | 15.5 | 14 | 93.59 | 374 |
| **3rdquartile** | 58 | 30 | 15 | 16.7 | 16 | 100 | 435 |
| **Maximum** | 62 | 40 | 17 | 18.3 | 17 | 100 | 544 |

**Appendix 7: Summary Statistics of Quantitative Traits in the M_2_ Population.**

DTF= Days to flowering, NOPPP= number of pods per plant, NOSPP= Number of seeds per pod, PL= Pod length, NOLP=Number of Locules per plant, PSST=Percentage Seed Set, NSPP=Number of seeds per Plant

|  | **DTF** | **NOPPP** | **NOSPP** | **PL** | **NOLP** | **PSST** | **NSPP** |
| --- | --- | --- | --- | --- | --- | --- | --- |
| **Minimum** | 38 | 1 | 0 | 8.3 | 4 | 0 | 0 |
| **1st quartile** | 41 | 7 | 12 | 16.1 | 14 | 84.63 | 96 |
| **Median** | 44 | 12 | 14 | 17.1 | 16 | 93.33 | 165 |
| **Mean** | 45 | 11 | 14 | 16.8 | 16 | 88.31 | 203 |
| **3rdquartile** | 47 | 19 | 16 | 17.8 | 17 | 100 | 272 |
| **Maximum** | 63 | 71 | 19 | 20.4 | 20 | 100 | 1156 |

# Appendix 8: Z-Test of Means of Number of Pods per Plant (NOPPP) Between the Wild type and Selected High Performing Putative Mutants Assuming Unequal Variances

|  | ***NOPPP*** | ***NOPPMUT*** |
| --- | --- | --- |
| Mean | 27.075 | 42.93069307 |
| Known Variance | 19.56392405 | 79.96514851 |
| Observations | 80 | 101 |
| Hypothesized Mean Difference | 0 |  |
| **z** | **-15.57564366** |  |
| P(Z<=z) one-tail | 0 |  |
| z Critical one-tail | 1.644853627 |  |
| P(Z<=z) two-tail | 0 |  |
| z Critical two-tail | 1.959963985 |  |

# Appendix 9: Z-Test of Means of Number of Seeds per Pod (NOSPP) Between the Wild type and Selected High Performing Putative Mutants Assuming Unequal Variances

|  | ***NOSPP*** | ***NOSPPMUT*** |
| --- | --- | --- |
| Mean | 13.75 | 18.093023 |
| Known Variance | 3.582278 | 0.085363 |
| Observations | 80 | 86 |
| Hypothesized Mean Difference | 0 |  |
| **z** | **-20.300028** |  |
| P(Z<=z) one-tail | 0 |  |
| z Critical one-tail | 1.6448536 |  |
| P(Z<=z) two-tail | 0 |  |
| z Critical two-tail | 1.959964 |  |

# Appendix 10: Z-Test of Means of Number of Locules per Pod (NOLPP) Between the Wild type and Selected High Performing Putative Mutants Assuming Unequal Variances

|  | ***NOLP*** | ***NOLPMUT*** |
| --- | --- | --- |
| Mean | 14.6875 | 18.262069 |
| Known Variance | 2.875791 | 0.242501 |
| Observations | 80 | 290 |
| Hypothesized Mean Difference | 0 |  |
| **z** | **-18.637883** |  |
| P(Z<=z) one-tail | 0 |  |
| z Critical one-tail | 1.6448536 |  |
| P(Z<=z) two-tail | 0 |  |
| z Critical two-tail | 1.959964 |  |

# Appendix 11: T-Test of Means of Pod Length (PL) Between the Wild type and Selected High Performing Putative Mutants Assuming Unequal Variances

|  | ***PL*** | ***PLMUT*** |
| --- | --- | --- |
| Mean | 15.45875 | 18.77316 |
| Known Variance | 2.881948 | 0.147885 |
| Observations | 80 | 231 |
| Hypothesized Mean Difference | 0 |  |
| **z** | **-17.309443** |  |
| P(Z<=z) one-tail | 0 |  |
| z Critical one-tail | 1.6448536 |  |
| P(Z<=z) two-tail | 0 |  |
| z Critical two-tail | 1.959964 |  |

# Appendix 12: T-Test of Means of Days to Flowering (DTF) Between the Wild type and Selected Early Flowering Performing Putative Mutants Assuming Unequal Variances

|  | ***DTF*** | ***DTFMUT*** |
| --- | --- | --- |
| Mean | 49.5125 | 39.66899 |
| Known Variance | 19.51883 | 1.042073 |
| Observations | 80 | 574 |
| Hypothesized Mean Difference | 0 |  |
| **z** | **19.854472** |  |
| P(Z<=z) one-tail | 0 |  |
| z Critical one-tail | 1.6448536 |  |
| P(Z<=z) two-tail | 0 |  |
| z Critical two-tail | 1.959964 |  |

# Appendix 13: T-Test of Means of Number of Seeds per Plant (NSPP) Between the Wild type and Selected High Performing Putative Mutants Assuming Unequal Variances

|  | ***NSPP*** | ***NSPPMUT*** |
| --- | --- | --- |
| Mean | 373.7 | 687.89412 |
| Known Variance | 7270.441 | 21731.24 |
| Observations | 80 | 85 |
| Hypothesized Mean Difference | 0 |  |
| **z** | **-16.877962** |  |
| P(Z<=z) one-tail | 0 |  |
| z Critical one-tail | 1.6448536 |  |
| P(Z<=z) two-tail | 0 |  |
| z Critical two-tail | 1.959964 |  |

# Appendix 14: T-Test of Means of Number of Pods per Plant (NOPPP) Between the Wild type and Selected Low Performing Putative Mutants Assuming Unequal Variances

|  | ***NOPPP*** | ***NOPPPMUT*** |
| --- | --- | --- |
| Mean | 27.075 | 5.562972292 |
| Known Variance | 19.56392405 | 6.35374173 |
| Observations | 80 | 794 |
| Hypothesized Mean Difference | 0 |  |
| **z** | **42.80619173** |  |
| P(Z<=z) one-tail | 0 |  |
| z Critical one-tail | 1.644853627 |  |
| P(Z<=z) two-tail | 0 |  |
| z Critical two-tail | 1.959963985 |  |

# Appendix 15: T-Test of Means of Number of Seeds per Pod (NOSPP) Between the Wild type and Selected Low Performing Putative Mutants Assuming Unequal Variances

|  | ***NOSPP*** | ***NOSPPMUT*** |
| --- | --- | --- |
| Mean | 13.75 | 5.584 |
| Known Variance | 3.582278 | 6.19651613 |
| Observations | 80 | 125 |
| Hypothesized Mean Difference | 0 |  |
| **z** | **26.58502** |  |
| P(Z<=z) one-tail | 0 |  |
| z Critical one-tail | 1.644854 |  |
| P(Z<=z) two-tail | 0 |  |
| z Critical two-tail | 1.959964 |  |

# Appendix 16: T-Test of Means of Number of Locules per Pod (NOLP) Between the Wild type and Selected Low Performing Putative Mutants Assuming Unequal Variances

|  | ***NOLP*** | ***NOLPMUT*** |
| --- | --- | --- |
| Mean | 14.6875 | 8.026316 |
| Known Variance | 2.875791 | 1.972262 |
| Observations | 80 | 38 |
| Hypothesized Mean Difference | 0 |  |
| **z** | **22.47414** |  |
| P(Z<=z) one-tail | 0 |  |
| z Critical one-tail | 1.644854 |  |
| P(Z<=z) two-tail | 0 |  |
| z Critical two-tail | 1.959964 |  |

# Appendix 17: T-Test of Means of Percentage Seed Set (PSST) Between the Wild type and Selected Low Performing Putative Mutants Assuming Unequal Variances

|  | ***PSST*** | ***PSSTMUT*** |
| --- | --- | --- |
| Mean | 93.50048 | 67.10391 |
| Known Variance | 34.21689 | 309.3106 |
| Observations | 80 | 426 |
| Hypothesized Mean Difference | 0 |  |
| **z** | **24.57444** |  |
| P(Z<=z) one-tail | 0 |  |
| z Critical one-tail | 1.644854 |  |
| P(Z<=z) two-tail | 0 |  |
| z Critical two-tail | 1.959964 |  |

# Appendix 18: T-Test of Means of Pod Length (PL) Between the Wild type and Selected Low Performing Putative Mutants Assuming Unequal Variances

|  | ***PL*** | ***PLMUT*** |
| --- | --- | --- |
| Mean | 15.45875 | 10.776 |
| Known Variance | 2.881948 | 1.345233 |
| Observations | 80 | 25 |
| Hypothesized Mean Difference | 0 |  |
| **z** | **15.62361** |  |
| P(Z<=z) one-tail | 0 |  |
| z Critical one-tail | 1.644854 |  |
| P(Z<=z) two-tail | 0 |  |
| z Critical two-tail | 1.959964 |  |

# Appendix 19: T-Test of Means of Days to Flowering (DTF) Between the Wild type and Selected Late Flowering Putative Mutants Assuming Unequal Variances

|  | ***DTF*** | ***DTFMUT*** |
| --- | --- | --- |
| Mean | 49.5125 | 61.70588 |
| Known Variance | 19.51883 | 0.470588 |
| Observations | 80 | 17 |
| Hypothesized Mean Difference | 0 |  |
| **z** | **-23.3941** |  |
| P(Z<=z) one-tail | 0 |  |
| z Critical one-tail | 1.644854 |  |
| P(Z<=z) two-tail | 0 |  |
| z Critical two-tail | 1.959964 |  |

# Appendix 20: Contrast analysis of high yielding mutants versus the wild type.

| **Traits** | **Plants** |  |  | **df** | **F** | **P>F** |
| --- | --- | --- | --- | --- | --- | --- |
| **Days to flowering** | C37P8 | vs | wild type | 1 | 7.64 | 0.0071 |
|  | C48P5 | vs | wild type | 1 | 7.64 | 0.0071 |
|  | C35P16 | vs | wild type | 1 | 7.64 | 0.0071 |
|  | C38P7 | vs | wild type | 1 | 7.64 | 0.0071 |
|  | B26P4 | vs | wild type | 1 | 7.64 | 0.0071 |
|  | D38P6 | vs | wild type | 1 | 7.64 | 0.0071 |
|  | C39P6 | vs | wild type | 1 | 7.64 | 0.0071 |
|  | D22P14 | vs | wild type | 1 | 7.64 | 0.0071 |
|  | C22P6 | vs | wild type | 1 | 7.64 | 0.0071 |
|  | C9P2 | vs | wild type | 1 | 7.64 | 0.0071 |
|  | E34P8 | vs | wild type | 1 | 7.64 | 0.0071 |
|  | B45P16 | vs | wild type | 1 | 7.64 | 0.0071 |
|  | C42P3 | vs | wild type | 1 | 7.64 | 0.0071 |
|  | D36P4 | vs | wild type | 1 | 7.64 | 0.0071 |
|  | D32P10 | vs | wild type | 1 | 7.64 | 0.0071 |
|  | B26P14 | vs | wild type | 1 | 7.64 | 0.0071 |
|  | B48P14 | vs | wild type | 1 | 7.64 | 0.0071 |
|  | C45P4 | vs | wild type | 1 | 7.64 | 0.0071 |
|  | C36P11 | vs | wild type | 1 | 7.64 | 0.0071 |
|  | C41P4 | vs | wild type | 1 | 7.64 | 0.0071 |
|  | C48P3 | vs | wild type | 1 | 7.64 | 0.0071 |
|  | C37P12 | vs | wild type | 1 | 7.64 | 0.0071 |
|  | C3P11 | vs | wild type | 1 | 7.64 | 0.0071 |
|  | D21P16 | vs | wild type | 1 | 7.64 | 0.0071 |
|  | C46P8 | vs | wild type | 1 | 7.64 | 0.0071 |
|  | C40P8 | vs | wild type | 1 | 7.64 | 0.0071 |
|  | C45P11 | vs | wild type | 1 | 7.64 | 0.0071 |

Appendix 20 cont’d

|  | B41P5 | vs | wild type | 1 | 7.64 | 0.0071 |
| --- | --- | --- | --- | --- | --- | --- |
|  | D37P2 | vs | wild type | 1 | 7.64 | 0.0071 |
|  | C40P9 | vs | wild type | 1 | 7.64 | 0.0071 |
|  | E26P2 | vs | wild type | 1 | 7.64 | 0.0071 |
|  | C45P1 | vs | wild type | 1 | 7.64 | 0.0071 |
|  | C21P9 | vs | wild type | 1 | 7.64 | 0.0071 |
|  | D32P14 | vs | wild type | 1 | 7.64 | 0.0071 |
|  | C42P8 | vs | wild type | 1 | 7.64 | 0.0071 |
|  | E33P14 | vs | wild type | 1 | 7.64 | 0.0071 |
|  | B50P9 | vs | wild type | 1 | 7.64 | 0.0071 |
|  | D28P15 | vs | wild type | 1 | 7.64 | 0.0071 |
|  | C33P4 | vs | wild type | 1 | 7.64 | 0.0071 |
|  | B52P13 | vs | wild type | 1 | 7.64 | 0.0071 |
|  | B44P9 | vs | wild type | 1 | 7.64 | 0.0071 |
|  | C39P5 | vs | wild type | 1 | 7.64 | 0.0071 |
|  | C19P5 | vs | wild type | 1 | 7.64 | 0.0071 |
|  | B26P16 | vs | wild type | 1 | 7.64 | 0.0071 |
|  | E31P9 | vs | wild type | 1 | 7.64 | 0.0071 |
|  | C29P10 | vs | wild type | 1 | 7.64 | 0.0071 |
|  | E33P15 | vs | wild type | 1 | 7.64 | 0.0071 |
|  | D26P16 | vs | wild type | 1 | 7.64 | 0.0071 |
|  | D36P3 | vs | wild type | 1 | 7.64 | 0.0071 |
|  | B40P3 | vs | wild type | 1 | 7.64 | 0.0071 |
|  | B55P15 | vs | wild type | 1 | 7.64 | 0.0071 |
|  | E49P7 | vs | wild type | 1 | 7.64 | 0.0071 |
|  | B44P14 | vs | wild type | 1 | 7.64 | 0.0071 |
|  | E33P1 | vs | wild type | 1 | 7.64 | 0.0071 |
|  | D41P2 | vs | wild type | 1 | 7.64 | 0.0071 |
|  | B48P12 | vs | wild type | 1 | 7.64 | 0.0071 |
|  | E34P1 | vs | wild type | 1 | 7.64 | 0.0071 |
|  | E34P2 | vs | wild type | 1 | 7.64 | 0.0071 |

Appendix 20 cont’d

|  | B48P10 | vs | wild type | 1 | 7.64 | 0.0071 |
| --- | --- | --- | --- | --- | --- | --- |
|  | D33P9 | vs | wild type | 1 | 7.64 | 0.0071 |
|  | C45P14 | vs | wild type | 1 | 7.64 | 0.0071 |
|  | C58P5 | vs | wild type | 1 | 7.64 | 0.0071 |
|  | D38P13 | vs | wild type | 1 | 7.64 | 0.0071 |
|  | E33P13 | vs | wild type | 1 | 7.64 | 0.0071 |
|  | E23P9 | vs | wild type | 1 | 7.64 | 0.0071 |
|  | E33P8 | vs | wild type | 1 | 7.64 | 0.0071 |
|  | B44P12 | vs | wild type | 1 | 7.64 | 0.0071 |
|  | E29P9 | vs | wild type | 1 | 7.64 | 0.0071 |
|  | E43P3 | vs | wild type | 1 | 7.64 | 0.0071 |
|  | D32P9 | vs | wild type | 1 | 7.64 | 0.0071 |
|  | C46P6 | vs | wild type | 1 | 7.64 | 0.0071 |
|  | B26P6 | vs | wild type | 1 | 7.64 | 0.0071 |
|  | C29P8 | vs | wild type | 1 | 7.64 | 0.0071 |
|  | C41P11 | vs | wild type | 1 | 7.64 | 0.0071 |
|  | C29P2 | vs | wild type | 1 | 7.64 | 0.0071 |
|  | E30P9 | vs | wild type | 1 | 7.64 | 0.0071 |
|  | C39P8 | vs | wild type | 1 | 7.64 | 0.0071 |
|  | D26P11 | vs | wild type | 1 | 7.64 | 0.0071 |
|  | E23P6 | vs | wild type | 1 | 7.64 | 0.0071 |
|  | D23P12 | vs | wild type | 1 | 7.64 | 0.0071 |
|  | B43P7 | vs | wild type | 1 | 7.64 | 0.0071 |
|  | C47P9 | vs | wild type | 1 | 7.64 | 0.0071 |
|  | E34P6 | vs | wild type | 1 | 7.64 | 0.0071 |
|  | B52P12 | vs | wild type | 1 | 7.64 | 0.0071 |
|  | B40P4 | vs | wild type | 1 | 7.64 | 0.0071 |
|  | C48P10 | vs | wild type | 1 | 7.64 | 0.0071 |
|  | D20P2 | vs | wild type | 1 | 7.64 | 0.0071 |
|  | B45P8 | vs | wild type | 1 | 7.64 | 0.0071 |
|  | B60P12 | vs | wild type | 1 | 7.64 | 0.0071 |
|  | C38P5 | vs | wild type | 1 | 7.64 | 0.0071 |
|  | E34P9 | vs | wild type | 1 | 7.64 | 0.0071 |
|  | C33P12 | vs | wild type | 1 | 7.64 | 0.0071 |
|  | B45P9 | vs | wild type | 1 | 7.64 | 0.0071 |
|  | E33P4 | vs | wild type | 1 | 7.64 | 0.0071 |
|  | B44P13 | vs | wild type | 1 | 7.64 | 0.0071 |
|  | C41P7 | vs | wild type | 1 | 7.64 | 0.0071 |
|  | E35P16 | vs | wild type | 1 | 7.64 | 0.0071 |
|  | E34P7 | vs | wild type | 1 | 7.64 | 0.0071 |

Appendix 20 cont’d

|  | C48P6 | vs | wild type | 1 | 7.64 | 0.0071 |
| --- | --- | --- | --- | --- | --- | --- |
|  | D48P14 | vs | wild type | 1 | 7.64 | 0.0071 |
|  | C27P6 | vs | wild type | 1 | 7.64 | 0.0071 |
|  | B55P3 | vs | wild type | 1 | 7.64 | 0.0071 |
|  | B57P18 | vs | wild type | 1 | 7.64 | 0.0071 |
|  | D26P8 | vs | wild type | 1 | 7.64 | 0.0071 |
|  | C34P16 | vs | wild type | 1 | 7.64 | 0.0071 |
|  | E33P7 | vs | wild type | 1 | 7.64 | 0.0071 |
|  | C41P2 | vs | wild type | 1 | 7.64 | 0.0071 |
|  | C22P14 | vs | wild type | 1 | 7.64 | 0.0071 |
|  | E29P14 | vs | wild type | 1 | 7.64 | 0.0071 |
|  | B44P3 | vs | wild type | 1 | 7.64 | 0.0071 |
|  | E35P3 | vs | wild type | 1 | 7.64 | 0.0071 |
|  | D34P1 | vs | wild type | 1 | 7.64 | 0.0071 |
|  | C33P2 | vs | wild type | 1 | 7.64 | 0.0071 |
|  | C28P3 | vs | wild type | 1 | 7.64 | 0.0071 |
|  | B41P17 | vs | wild type | 1 | 7.64 | 0.0071 |
|  | E42P9 | vs | wild type | 1 | 7.64 | 0.0071 |
|  | D20P4 | vs | wild type | 1 | 7.64 | 0.0071 |
|  | B41P3 | vs | wild type | 1 | 7.64 | 0.0071 |
|  | E28P14 | vs | wild type | 1 | 7.64 | 0.0071 |
|  | C49P11 | vs | wild type | 1 | 7.64 | 0.0071 |
|  | C57P11 | vs | wild type | 1 | 7.64 | 0.0071 |
|  | C38P11 | vs | wild type | 1 | 7.64 | 0.0071 |
|  | B41P10 | vs | wild type | 1 | 7.64 | 0.0071 |
|  | C48P1 | vs | wild type | 1 | 7.64 | 0.0071 |
|  | E33P3 | vs | wild type | 1 | 7.64 | 0.0071 |
|  | C55P9 | vs | wild type | 1 | 7.64 | 0.0071 |
|  | D45P4 | vs | wild type | 1 | 7.64 | 0.0071 |
|  | E30P16 | vs | wild type | 1 | 7.64 | 0.0071 |
|  | D47P4 | vs | wild type | 1 | 7.64 | 0.0071 |
|  | D38P7 | vs | wild type | 1 | 7.64 | 0.0071 |
|  | E33P5 | vs | wild type | 1 | 7.64 | 0.0071 |
|  | D53P4 | vs | wild type | 1 | 7.64 | 0.0071 |
|  | B41P6 | vs | wild type | 1 | 7.64 | 0.0071 |
|  | D37P6 | vs | wild type | 1 | 7.64 | 0.0071 |
|  | D34P6 | vs | wild type | 1 | 7.64 | 0.0071 |
|  | C49P6 | vs | wild type | 1 | 7.64 | 0.0071 |
|  | B44P6 | vs | wild type | 1 | 7.64 | 0.0071 |
|  | C49P1 | vs | wild type | 1 | 7.64 | 0.0071 |

Appendix 20 cont’d

|  | E38P4 | vs | wild type | 1 | 7.64 | 0.0071 |
| --- | --- | --- | --- | --- | --- | --- |
|  | E30P14 | vs | wild type | 1 | 7.64 | 0.0071 |
|  | E35P5 | vs | wild type | 1 | 7.64 | 0.0071 |
|  | C47P7 | vs | wild type | 1 | 7.64 | 0.0071 |
|  | E33P2 | vs | wild type | 1 | 7.64 | 0.0071 |
|  | B44P2 | vs | wild type | 1 | 7.64 | 0.0071 |
|  | C9P1 | vs | wild type | 1 | 7.64 | 0.0071 |
|  | E52P17 | vs | wild type | 1 | 7.64 | 0.0071 |
|  | C58P1 | vs | wild type | 1 | 7.64 | 0.0071 |
|  | E44P5 | vs | wild type | 1 | 7.64 | 0.0071 |
|  | B48P11 | vs | wild type | 1 | 7.64 | 0.0071 |
|  | C20P9 | vs | wild type | 1 | 7.64 | 0.0071 |
|  | B57P11 | vs | wild type | 1 | 7.64 | 0.0071 |
|  | B58P6 | vs | wild type | 1 | 7.64 | 0.0071 |
|  | E24P18 | vs | wild type | 1 | 7.64 | 0.0071 |
|  | C42P1 | vs | wild type | 1 | 7.64 | 0.0071 |
|  | E49P5 | vs | wild type | 1 | 7.64 | 0.0071 |
|  | E30P12 | vs | wild type | 1 | 7.64 | 0.0071 |
|  | C32P7 | vs | wild type | 1 | 7.64 | 0.0071 |
|  | E34P16 | vs | wild type | 1 | 7.64 | 0.0071 |
|  | E33P11 | vs | wild type | 1 | 7.64 | 0.0071 |
|  | C35P7 | vs | wild type | 1 | 7.64 | 0.0071 |
|  | D26P7 | vs | wild type | 1 | 7.64 | 0.0071 |
|  | E52P12 | vs | wild type | 1 | 7.64 | 0.0071 |
|  | E36P16 | vs | wild type | 1 | 7.64 | 0.0071 |
|  | D32P2 | vs | wild type | 1 | 7.64 | 0.0071 |
|  | E36P7 | vs | wild type | 1 | 8.71 | 0.0042 |
|  | D32P15 | vs | wild type | 1 | 8.71 | 0.0042 |
|  | E34P10 | vs | wild type | 1 | 8.71 | 0.0042 |
|  | D35P12 | vs | wild type | 1 | 8.71 | 0.0042 |
|  | C36P14 | vs | wild type | 1 | 8.71 | 0.0042 |
|  | C49P9 | vs | wild type | 1 | 8.71 | 0.0042 |
|  | E33P9 | vs | wild type | 1 | 8.71 | 0.0042 |
|  | D35P14 | vs | wild type | 1 | 8.71 | 0.0042 |
|  | E34P11 | vs | wild type | 1 | 8.71 | 0.0042 |
|  | C34P12 | vs | wild type | 1 | 8.71 | 0.0042 |
|  | C47P15 | vs | wild type | 1 | 8.71 | 0.0042 |
|  | E30P8 | vs | wild type | 1 | 8.71 | 0.0042 |
|  | C41P1 | vs | wild type | 1 | 8.71 | 0.0042 |
|  | E29P8 | vs | wild type | 1 | 8.71 | 0.0042 |
|  | E35P13 | vs | wild type | 1 | 8.71 | 0.0042 |

Appendix 20 cont’d

|  | D38P4 | vs | wild type | 1 | 8.71 | 0.0042 |
| --- | --- | --- | --- | --- | --- | --- |
|  | D38P8 | vs | wild type | 1 | 8.71 | 0.0042 |
|  | C34P9 | vs | wild type | 1 | 8.71 | 0.0042 |
|  | C36P12 | vs | wild type | 1 | 8.71 | 0.0042 |
|  | E32P6 | vs | wild type | 1 | 8.71 | 0.0042 |
|  | C34P14 | vs | wild type | 1 | 8.71 | 0.0042 |
|  | C17P4 | vs | wild type | 1 | 8.71 | 0.0042 |
|  | C44P9 | vs | wild type | 1 | 8.71 | 0.0042 |
|  | C36P8 | vs | wild type | 1 | 8.71 | 0.0042 |
|  | C25P9 | vs | wild type | 1 | 8.71 | 0.0042 |
|  | D37P3 | vs | wild type | 1 | 8.71 | 0.0042 |
|  | D32P17 | vs | wild type | 1 | 8.71 | 0.0042 |
|  | C37P5 | vs | wild type | 1 | 8.71 | 0.0042 |
|  | D31P15 | vs | wild type | 1 | 8.71 | 0.0042 |
|  | C38P3 | vs | wild type | 1 | 8.71 | 0.0042 |
|  | C21P7 | vs | wild type | 1 | 8.71 | 0.0042 |
|  | C36P13 | vs | wild type | 1 | 8.71 | 0.0042 |
|  | E35P14 | vs | wild type | 1 | 8.71 | 0.0042 |
|  | D23P10 | vs | wild type | 1 | 8.71 | 0.0042 |
|  | D32P18 | vs | wild type | 1 | 8.71 | 0.0042 |
|  | C46P11 | vs | wild type | 1 | 8.71 | 0.0042 |
|  | C20P2 | vs | wild type | 1 | 8.71 | 0.0042 |
|  | E36P8 | vs | wild type | 1 | 8.71 | 0.0042 |
|  | E30P7 | vs | wild type | 1 | 8.71 | 0.0042 |
|  | D38P2 | vs | wild type | 1 | 8.71 | 0.0042 |
|  | B41P18 | vs | wild type | 1 | 8.71 | 0.0042 |
|  | E35P6 | vs | wild type | 1 | 8.71 | 0.0042 |
|  | C23P3 | vs | wild type | 1 | 8.71 | 0.0042 |
|  | D38P1 | vs | wild type | 1 | 8.71 | 0.0042 |
|  | D32P19 | vs | wild type | 1 | 8.71 | 0.0042 |
|  | D35P9 | vs | wild type | 1 | 8.71 | 0.0042 |
|  | C37P13 | vs | wild type | 1 | 8.71 | 0.0042 |
|  | D35P10 | vs | wild type | 1 | 8.71 | 0.0042 |
|  | C19P15 | vs | wild type | 1 | 8.71 | 0.0042 |
|  | C25P10 | vs | wild type | 1 | 8.71 | 0.0042 |
|  | E28P1 | vs | wild type | 1 | 8.71 | 0.0042 |
|  | B43P2 | vs | wild type | 1 | 8.71 | 0.0042 |
|  | E38P1 | vs | wild type | 1 | 8.71 | 0.0042 |
|  | D41P8 | vs | wild type | 1 | 8.71 | 0.0042 |
|  | C47P6 | vs | wild type | 1 | 8.71 | 0.0042 |
|  | E32P5 | vs | wild type | 1 | 8.71 | 0.0042 |

Appendix 20 cont’d

|  | E29P7 | vs | wild type | 1 | 8.71 | 0.0042 |
| --- | --- | --- | --- | --- | --- | --- |
|  | D34P2 | vs | wild type | 1 | 8.71 | 0.0042 |
|  | B43P4 | vs | wild type | 1 | 8.71 | 0.0042 |
|  | E35P8 | vs | wild type | 1 | 8.71 | 0.0042 |
|  | C59P7 | vs | wild type | 1 | 8.71 | 0.0042 |
|  | E32P2 | vs | wild type | 1 | 8.71 | 0.0042 |
|  | C34P13 | vs | wild type | 1 | 8.71 | 0.0042 |
|  | E32P7 | vs | wild type | 1 | 8.71 | 0.0042 |
|  | D36P2 | vs | wild type | 1 | 8.71 | 0.0042 |
|  | D38P5 | vs | wild type | 1 | 8.71 | 0.0042 |
|  | C29P3 | vs | wild type | 1 | 8.71 | 0.0042 |
|  | E36P2 | vs | wild type | 1 | 8.71 | 0.0042 |
|  | C37P6 | vs | wild type | 1 | 8.71 | 0.0042 |
|  | D41P1 | vs | wild type | 1 | 8.71 | 0.0042 |
|  | C38P2 | vs | wild type | 1 | 8.71 | 0.0042 |
|  | D28P12 | vs | wild type | 1 | 8.71 | 0.0042 |
|  | C46P7 | vs | wild type | 1 | 8.71 | 0.0042 |
|  | D35P8 | vs | wild type | 1 | 8.71 | 0.0042 |
|  | E36P14 | vs | wild type | 1 | 8.71 | 0.0042 |
|  | E37P4 | vs | wild type | 1 | 8.71 | 0.0042 |
|  | D41P9 | vs | wild type | 1 | 8.71 | 0.0042 |
|  | B57P10 | vs | wild type | 1 | 8.71 | 0.0042 |
|  | C36P9 | vs | wild type | 1 | 8.71 | 0.0042 |
|  | E35P10 | vs | wild type | 1 | 8.71 | 0.0042 |
|  | D41P7 | vs | wild type | 1 | 8.71 | 0.0042 |
|  | E33P12 | vs | wild type | 1 | 8.71 | 0.0042 |
|  | E35P9 | vs | wild type | 1 | 8.71 | 0.0042 |
|  | C35P8 | vs | wild type | 1 | 8.71 | 0.0042 |
|  | E33P10 | vs | wild type | 1 | 8.71 | 0.0042 |
|  | C36P10 | vs | wild type | 1 | 8.71 | 0.0042 |
|  | C21P11 | vs | wild type | 1 | 8.71 | 0.0042 |
| **PL** | C50P2 | vs | wild type | 1 | 7.12 | 0.0092 |
|  | D24P4 | vs | wild type | 1 | 7.76 | 0.0067 |
|  | D54P10 | vs | wild type | 1 | 7.76 | 0.0067 |
|  | C58P13 | vs | wild type | 1 | 9.12 | 0.0034 |
|  | D32P2 | vs | wild type | 1 | 9.84 | 0.0024 |
|  | C54P7 | vs | wild type | 1 | 10.97 | 0.0014 |
|  | C21P11 | vs | wild type | 1 | 12.58 | 0.0007 |
|  | C37P1 | vs | wild type | 1 | 13.86 | 0.0004 |
|  | D62P1 | vs | wild type | 1 | 15.2 | 0.0002 |
|  | A64P11 | vs | wild type | 1 | 15.65 | 0.0002 |
|  | E55P1 | vs | wild type | 1 | 17.56 | 0.0001 |

Appendix 20 cont’d

| **NOLP** | C36P4 | vs | wild type | 1 | 10.37 | 0.0019 |
| --- | --- | --- | --- | --- | --- | --- |
|  | C36P14 | vs | wild type | 1 | 10.37 | 0.0019 |
|  | C33P8 | vs | wild type | 1 | 10.37 | 0.0019 |
|  | D29P9 | vs | wild type | 1 | 10.37 | 0.0019 |
|  | E50P2 | vs | wild type | 1 | 10.37 | 0.0019 |
|  | A64P7 | vs | wild type | 1 | 10.37 | 0.0019 |
|  | D33P8 | vs | wild type | 1 | 10.37 | 0.0019 |
| **NOSP** | E60P4 | vs | wild type | 1 | 7.6 | 0.0072 |
|  | D41P12 | vs | wild type | 1 | 7.6 | 0.0072 |
|  | B26P8 | vs | wild type | 1 | 7.6 | 0.0072 |
|  | C35P8 | vs | wild type | 1 | 7.6 | 0.0072 |
|  | C44P5 | vs | wild type | 1 | 7.6 | 0.0072 |
|  | C20P6 | vs | wild type | 1 | 7.6 | 0.0072 |
|  | C21P12 | vs | wild type | 1 | 7.6 | 0.0072 |
|  | C39P6 | vs | wild type | 1 | 7.6 | 0.0072 |
| **NSPP** | A57P1 | vs | wild type | 1 | 7.08 | 0.0094 |
|  | A57P2 | vs | wild type | 1 | 7.08 | 0.0094 |
|  | C33P2 | vs | wild type | 1 | 7.08 | 0.0094 |
|  | C20P6 | vs | wild type | 1 | 7.46 | 0.0078 |
|  | C45P11 | vs | wild type | 1 | 7.46 | 0.0078 |
|  | D38P4 | vs | wild type | 1 | 7.46 | 0.0078 |
|  | E43P3 | vs | wild type | 1 | 7.46 | 0.0078 |
|  | A55P1 | vs | wild type | 1 | 7.71 | 0.0068 |
|  | C31P1 | vs | wild type | 1 | 7.71 | 0.0068 |
|  | C33P3 | vs | wild type | 1 | 7.71 | 0.0068 |
|  | D28P15 | vs | wild type | 1 | 7.71 | 0.0068 |
|  | D44P14 | vs | wild type | 1 | 7.91 | 0.0062 |
|  | C21P12 | vs | wild type | 1 | 8.72 | 0.0042 |
|  | C25P2 | vs | wild type | 1 | 8.72 | 0.0042 |
|  | B26P4 | vs | wild type | 1 | 8.85 | 0.0039 |
|  | C38P6 | vs | wild type | 1 | 8.85 | 0.0039 |
|  | D36P4 | vs | wild type | 1 | 8.85 | 0.0039 |
|  | D38P6 | vs | wild type | 1 | 8.85 | 0.0039 |
|  | D20P11 | vs | wild type | 1 | 8.92 | 0.0037 |
|  | D33P4 | vs | wild type | 1 | 8.92 | 0.0037 |
|  | E35P12 | vs | wild type | 1 | 8.92 | 0.0037 |
|  | C37P7 | vs | wild type | 1 | 10.22 | 0.0020 |
|  | C42P7 | vs | wild type | 1 | 10.22 | 0.0020 |
|  | D35P12 | vs | wild type | 1 | 10.22 | 0.0020 |
|  | C28P1 | vs | wild type | 1 | 10.83 | 0.0015 |
|  | D24P3 | vs | wild type | 1 | 11.13 | 0.0013 |
|  | D26P1 | vs | wild type | 1 | 11.13 | 0.0013 |
|  | D35P14 | vs | wild type | 1 | 11.37 | 0.0012 |

Appendix 20 cont’d

|  | D28P2 | vs | wild type | 1 | 11.61 | 0.0010 |
| --- | --- | --- | --- | --- | --- | --- |
|  | D24P1 | vs | wild type | 1 | 12.09 | 0.0008 |
|  | D44P15 | vs | wild type | 1 | 12.09 | 0.0008 |
|  | E28P14 | vs | wild type | 1 | 12.09 | 0.0008 |
|  | D37P5 | vs | wild type | 1 | 12.41 | 0.0007 |
|  | C39P6 | vs | wild type | 1 | 13.08 | 0.0005 |
|  | C35P5 | vs | wild type | 1 | 13.08 | 0.0005 |
|  | C38P4 | vs | wild type | 1 | 13.08 | 0.0005 |
|  | C46P12 | vs | wild type | 1 | 13.08 | 0.0005 |
|  | E36P10 | vs | wild type | 1 | 13.25 | 0.0005 |
|  | C27P10 | vs | wild type | 1 | 13.59 | 0.0004 |
|  | C36P8 | vs | wild type | 1 | 14.2 | 0.0003 |
|  | D37P3 | vs | wild type | 1 | 14.91 | 0.0002 |
|  | E27P8 | vs | wild type | 1 | 17.05 | 0.0001 |
|  | C33P13 | vs | wild type | 1 | 17.34 | 0.0001 |
|  | C3P11 | vs | wild type | 1 | 19.85 | 0.0000 |
|  | C20P3 | vs | wild type | 1 | 20.8 | 0.0000 |
|  | B26P6 | vs | wild type | 1 | 24.69 | 0.0000 |
|  | C29P10 | vs | wild type | 1 | 26.58 | 0.0000 |
|  | E26P8 | vs | wild type | 1 | 28.04 | 0.0000 |
|  | C41P6 | vs | wild type | 1 | 28.66 | 0.0000 |
|  | C36P12 | vs | wild type | 1 | 29.54 | 0.0000 |
|  | C20P2 | vs | wild type | 1 | 35.37 | 0.0000 |
|  | E32P6 | vs | wild type | 1 | 39.07 | 0.0000 |
|  | C39P1 | vs | wild type | 1 | 46.7 | 0.0000 |
|  | D32P18 | vs | wild type | 1 | 46.7 | 0.0000 |
|  | E26P3 | vs | wild type | 1 | 47.66 | 0.0000 |
|  | C37P4 | vs | wild type | 1 | 48.63 | 0.0000 |
|  | C21P8 | vs | wild type | 1 | 57.45 | 0.0000 |
|  | C41P8 | vs | wild type | 1 | 64.92 | 0.0000 |
|  | C37P8 | vs | wild type | 1 | 78.53 | 0.0000 |
|  | C37P9 | vs | wild type | 1 | 82.29 | 0.0000 |
|  | C37P12 | vs | wild type | 1 | 83.14 | 0.0000 |
| NOPPP | D35P14 | vs | wild type | 1 | 7.18 | 0.0090 |
|  | D36P1 | vs | wild type | 1 | 7.18 | 0.0090 |
|  | D36P15 | vs | wild type | 1 | 7.18 | 0.0090 |
|  | E25P5 | vs | wild type | 1 | 7.18 | 0.0090 |
|  | D33P1 | vs | wild type | 1 | 8.43 | 0.0048 |
|  | E28P9 | vs | wild type | 1 | 8.43 | 0.0048 |
|  | E32P10 | vs | wild type | 1 | 8.43 | 0.0048 |
|  | E32P11 | vs | wild type | 1 | 8.43 | 0.0048 |
|  | E33P1 | vs | wild type | 1 | 8.43 | 0.0048 |
|  | C28P1 | vs | wild type | 1 | 9.79 | 0.0025 |

Appendix 20 cont’d

|  | C36P8 | vs | wild type | 1 | 9.79 | 0.0025 |
| --- | --- | --- | --- | --- | --- | --- |
|  | D44P14 | vs | wild type | 1 | 9.79 | 0.0025 |
|  | D55P2 | vs | wild type | 1 | 9.79 | 0.0025 |
|  | E33P6 | vs | wild type | 1 | 9.79 | 0.0025 |
|  | C3P11 | vs | wild type | 1 | 11.25 | 0.0012 |
|  | C41P11 | vs | wild type | 1 | 11.25 | 0.0012 |
|  | D44P6 | vs | wild type | 1 | 11.25 | 0.0012 |
|  | E36P11 | vs | wild type | 1 | 11.25 | 0.0012 |
|  | A57P1 | vs | wild type | 1 | 12.8 | 0.0006 |
|  | A57P2 | vs | wild type | 1 | 12.8 | 0.0006 |
|  | B55P10 | vs | wild type | 1 | 12.8 | 0.0006 |
|  | C33P13 | vs | wild type | 1 | 12.8 | 0.0006 |
|  | C33P2 | vs | wild type | 1 | 12.8 | 0.0006 |
|  | D36P13 | vs | wild type | 1 | 12.8 | 0.0006 |
|  | D24P3 | vs | wild type | 1 | 14.46 | 0.0003 |
|  | D26P1 | vs | wild type | 1 | 14.46 | 0.0003 |
|  | C20P3 | vs | wild type | 1 | 16.22 | 0.0001 |
|  | C25P3 | vs | wild type | 1 | 16.22 | 0.0001 |
|  | E35P12 | vs | wild type | 1 | 16.22 | 0.0001 |
|  | C27P10 | vs | wild type | 1 | 18.08 | 0.0001 |
|  | D34P10 | vs | wild type | 1 | 18.08 | 0.0001 |
|  | E26P8 | vs | wild type | 1 | 18.08 | 0.0001 |
|  | D37P3 | vs | wild type | 1 | 20.04 | 0.0000 |
|  | C29P10 | vs | wild type | 1 | 22.1 | 0.0000 |
|  | D24P1 | vs | wild type | 1 | 22.1 | 0.0000 |
|  | C41P6 | vs | wild type | 1 | 24.27 | 0.0000 |
|  | E36P10 | vs | wild type | 1 | 24.27 | 0.0000 |
|  | B26P6 | vs | wild type | 1 | 26.53 | 0.0000 |
|  | C20P2 | vs | wild type | 1 | 31.36 | 0.0000 |
|  | D37P5 | vs | wild type | 1 | 31.36 | 0.0000 |
|  | C37P4 | vs | wild type | 1 | 36.6 | 0.0000 |
|  | C36P12 | vs | wild type | 1 | 42.24 | 0.0000 |
|  | D44P15 | vs | wild type | 1 | 42.24 | 0.0000 |
|  | E27P8 | vs | wild type | 1 | 42.24 | 0.0000 |
|  | E28P14 | vs | wild type | 1 | 42.24 | 0.0000 |
|  | C25P2 | vs | wild type | 1 | 45.21 | 0.0000 |
|  | C39P1 | vs | wild type | 1 | 54.73 | 0.0000 |
|  | D32P18 | vs | wild type | 1 | 54.73 | 0.0000 |
|  | C37P8 | vs | wild type | 1 | 65.15 | 0.0000 |
|  | C21P8 | vs | wild type | 1 | 68.83 | 0.0000 |
|  | C37P9 | vs | wild type | 1 | 68.83 | 0.0000 |
|  | E32P6 | vs | wild type | 1 | 72.61 | 0.0000 |
|  | C37P12 | vs | wild type | 1 | 84.55 | 0.0000 |
|  | E26P3 | vs | wild type | 1 | 88.74 | 0.0000 |
|  | C41P8 | vs | wild type | 1 | 97.4 | 0.0000 |

# Appendix 21: Contrast analysis of low yielding mutants versus the wild type.

| **Traits** |  |  |  | **df** | **F** | **P>F** |
| --- | --- | --- | --- | --- | --- | --- |
| PL | C47P2 | vs | wild type | 1 | 8.37 | 0.0049 |
|  | C37P2 | vs | wild type | 1 | 7.7 | 0.0069 |
|  | C44P5 | vs | wild type | 1 | 7.38 | 0.0081 |
| PSST | D32P1 | vs | wild type | 1 | 252.34 | 0.0000 |
|  | E55P11 | vs | wild type | 1 | 252.34 | 0.0000 |
|  | E57P17 | vs | wild type | 1 | 252.34 | 0.0000 |
|  | E31P5 | vs | wild type | 1 | 252.34 | 0.0000 |
|  | E46P10 | vs | wild type | 1 | 252.34 | 0.0000 |
|  | E43P13 | vs | wild type | 1 | 252.34 | 0.0000 |
|  | D24P4 | vs | wild type | 1 | 252.34 | 0.0000 |
|  | D60P6 | vs | wild type | 1 | 219.74 | 0.0000 |
|  | A61P9 | vs | wild type | 1 | 217.63 | 0.0000 |
|  | D60P5 | vs | wild type | 1 | 217.63 | 0.0000 |
|  | B58P13 | vs | wild type | 1 | 217.63 | 0.0000 |
|  | E57P11 | vs | wild type | 1 | 209.38 | 0.0000 |
|  | E57P8 | vs | wild type | 1 | 195.94 | 0.0000 |
|  | D61P6 | vs | wild type | 1 | 185.52 | 0.0000 |
|  | E43P16 | vs | wild type | 1 | 176.16 | 0.0000 |
|  | E43P2 | vs | wild type | 1 | 161.29 | 0.0000 |
|  | D44P18 | vs | wild type | 1 | 149.93 | 0.0000 |
|  | D32P2 | vs | wild type | 1 | 146.66 | 0.0000 |
|  | E57P2 | vs | wild type | 1 | 146.66 | 0.0000 |
|  | E48P9 | vs | wild type | 1 | 135.44 | 0.0000 |
|  | D44P3 | vs | wild type | 1 | 128.92 | 0.0000 |
|  | C61P7 | vs | wild type | 1 | 126.61 | 0.0000 |
|  | B63P6 | vs | wild type | 1 | 126.61 | 0.0000 |
|  | D21P7 | vs | wild type | 1 | 121.69 | 0.0000 |
|  | B64P7 | vs | wild type | 1 | 113.59 | 0.0000 |
|  | E42P13 | vs | wild type | 1 | 111.85 | 0.0000 |
|  | D44P2 | vs | wild type | 1 | 111.85 | 0.0000 |
|  | D38P7 | vs | wild type | 1 | 104.5 | 0.0000 |
|  | E49P4 | vs | wild type | 1 | 104.5 | 0.0000 |
|  | D62P1 | vs | wild type | 1 | 104.5 | 0.0000 |
|  | C25P12 | vs | wild type | 1 | 94.24 | 0.0000 |
|  | E49P14 | vs | wild type | 1 | 94.24 | 0.0000 |
|  | D25P10 | vs | wild type | 1 | 82.62 | 0.0000 |
|  | D46P6 | vs | wild type | 1 | 82.62 | 0.0000 |
|  | C20P13 | vs | wild type | 1 | 82.62 | 0.0000 |

Appendix 21 cont’d

|  | D26P5 | vs | wild type | 1 | 79.02 | 0.0000 |
| --- | --- | --- | --- | --- | --- | --- |
|  | D22P11 | vs | wild type | 1 | 77.54 | 0.0000 |
|  | E43P9 | vs | wild type | 1 | 74.02 | 0.0000 |
|  | E35P9 | vs | wild type | 1 | 74.02 | 0.0000 |
|  | E40P4 | vs | wild type | 1 | 74.02 | 0.0000 |
|  | E59P7 | vs | wild type | 1 | 69.48 | 0.0000 |
|  | D22P10 | vs | wild type | 1 | 69.48 | 0.0000 |
|  | C54P7 | vs | wild type | 1 | 69.48 | 0.0000 |
|  | E23P2 | vs | wild type | 1 | 69.48 | 0.0000 |
|  | E48P10 | vs | wild type | 1 | 69.48 | 0.0000 |
|  | D43P12 | vs | wild type | 1 | 66.64 | 0.0000 |
|  | D22P15 | vs | wild type | 1 | 66.64 | 0.0000 |
|  | E49P10 | vs | wild type | 1 | 66.64 | 0.0000 |
|  | E41P8 | vs | wild type | 1 | 63.3 | 0.0000 |
|  | D25P5 | vs | wild type | 1 | 62.25 | 0.0000 |
|  | A64P8 | vs | wild type | 1 | 54.62 | 0.0000 |
|  | A59P7 | vs | wild type | 1 | 54.62 | 0.0000 |
|  | D50P14 | vs | wild type | 1 | 54.62 | 0.0000 |
|  | E58P7 | vs | wild type | 1 | 54.62 | 0.0000 |
|  | B58P5 | vs | wild type | 1 | 54.62 | 0.0000 |
|  | E45P8 | vs | wild type | 1 | 54.62 | 0.0000 |
|  | E46P1 | vs | wild type | 1 | 54.62 | 0.0000 |
|  | E51P7 | vs | wild type | 1 | 54.62 | 0.0000 |
|  | D54P10 | vs | wild type | 1 | 54.62 | 0.0000 |
|  | A59P6 | vs | wild type | 1 | 47.49 | 0.0000 |
|  | B52P10 | vs | wild type | 1 | 47.49 | 0.0000 |
|  | C22P13 | vs | wild type | 1 | 46.58 | 0.0000 |
|  | B54P7 | vs | wild type | 1 | 46.58 | 0.0000 |
|  | E55P4 | vs | wild type | 1 | 46.58 | 0.0000 |
|  | E36P9 | vs | wild type | 1 | 46.58 | 0.0000 |
|  | E36P12 | vs | wild type | 1 | 46.58 | 0.0000 |
|  | E53P12 | vs | wild type | 1 | 45.38 | 0.0000 |
|  | E42P8 | vs | wild type | 1 | 45.38 | 0.0000 |
|  | A64P11 | vs | wild type | 1 | 43.79 | 0.0000 |
|  | E51P16 | vs | wild type | 1 | 43.79 | 0.0000 |
|  | E24P12 | vs | wild type | 1 | 43.79 | 0.0000 |
|  | E34P16 | vs | wild type | 1 | 43.79 | 0.0000 |
|  | E37P3 | vs | wild type | 1 | 41.55 | 0.0000 |
|  | D63P9 | vs | wild type | 1 | 40.05 | 0.0000 |
|  | A48P12 | vs | wild type | 1 | 40.05 | 0.0000 |
|  | D22P2 | vs | wild type | 1 | 40.05 | 0.0000 |

Appendix 21 cont’d

|  | A48P13 | vs | wild type | 1 | 40.05 | 0.0000 |
| --- | --- | --- | --- | --- | --- | --- |
|  | E31P1 | vs | wild type | 1 | 40.05 | 0.0000 |
|  | D61P1 | vs | wild type | 1 | 38.16 | 0.0000 |
|  | E43P15 | vs | wild type | 1 | 38.16 | 0.0000 |
|  | E51P13 | vs | wild type | 1 | 38.16 | 0.0000 |
|  | E49P5 | vs | wild type | 1 | 38.16 | 0.0000 |
|  | D28P7 | vs | wild type | 1 | 35.7 | 0.0000 |
|  | E57P16 | vs | wild type | 1 | 35.7 | 0.0000 |
|  | E24P15 | vs | wild type | 1 | 35.7 | 0.0000 |
|  | C20P5 | vs | wild type | 1 | 35.7 | 0.0000 |
|  | C61P11 | vs | wild type | 1 | 34.72 | 0.0000 |
|  | E59P1 | vs | wild type | 1 | 34.72 | 0.0000 |
|  | D21P17 | vs | wild type | 1 | 34.72 | 0.0000 |
|  | D46P3 | vs | wild type | 1 | 34.72 | 0.0000 |
|  | E39P2 | vs | wild type | 1 | 34.72 | 0.0000 |
|  | A64P7 | vs | wild type | 1 | 32.39 | 0.0000 |
|  | D33P8 | vs | wild type | 1 | 32.39 | 0.0000 |
|  | C56P9 | vs | wild type | 1 | 32.39 | 0.0000 |
|  | D37P6 | vs | wild type | 1 | 32.39 | 0.0000 |
|  | E59P3 | vs | wild type | 1 | 32.39 | 0.0000 |
|  | E45P2 | vs | wild type | 1 | 32.39 | 0.0000 |
|  | E49P3 | vs | wild type | 1 | 32.39 | 0.0000 |
|  | E53P14 | vs | wild type | 1 | 29.48 | 0.0000 |
|  | C60P6 | vs | wild type | 1 | 29.48 | 0.0000 |
|  | D1P6 | vs | wild type | 1 | 29.48 | 0.0000 |
|  | C48P2 | vs | wild type | 1 | 29.48 | 0.0000 |
|  | D30P8 | vs | wild type | 1 | 29.48 | 0.0000 |
|  | E41P3 | vs | wild type | 1 | 27.74 | 0.0000 |
|  | E43P14 | vs | wild type | 1 | 27.74 | 0.0000 |
|  | C32P5 | vs | wild type | 1 | 27.74 | 0.0000 |
|  | D31P14 | vs | wild type | 1 | 27.74 | 0.0000 |
|  | D44P4 | vs | wild type | 1 | 27.74 | 0.0000 |
|  | E49P15 | vs | wild type | 1 | 27.74 | 0.0000 |
|  | D35P4 | vs | wild type | 1 | 27.74 | 0.0000 |
|  | E41P14 | vs | wild type | 1 | 27.74 | 0.0000 |
|  | D20P8 | vs | wild type | 1 | 27.74 | 0.0000 |
|  | C64P3 | vs | wild type | 1 | 25.74 | 0.0000 |
|  | E50P14 | vs | wild type | 1 | 25.74 | 0.0000 |
|  | E30P12 | vs | wild type | 1 | 25.74 | 0.0000 |
|  | B51P8 | vs | wild type | 1 | 24.63 | 0.0000 |

Appendix 21 cont’d

|  | B64P12 | vs | wild type | 1 | 24.63 | 0.0000 |
| --- | --- | --- | --- | --- | --- | --- |
|  | E41P9 | vs | wild type | 1 | 24.63 | 0.0000 |
|  | E56P5 | vs | wild type | 1 | 24.63 | 0.0000 |
|  | E35P10 | vs | wild type | 1 | 24.63 | 0.0000 |
|  | B41P9 | vs | wild type | 1 | 24.63 | 0.0000 |
|  | E26P1 | vs | wild type | 1 | 24.63 | 0.0000 |
|  | B47P6 | vs | wild type | 1 | 23.93 | 0.0000 |
|  | D29P1 | vs | wild type | 1 | 23.93 | 0.0000 |
|  | E24P14 | vs | wild type | 1 | 23.93 | 0.0000 |
|  | E36P7 | vs | wild type | 1 | 23.93 | 0.0000 |
|  | E59P5 | vs | wild type | 1 | 20.78 | 0.0000 |
|  | A52P6 | vs | wild type | 1 | 20.78 | 0.0000 |
|  | A63P10 | vs | wild type | 1 | 20.78 | 0.0000 |
|  | A47P12 | vs | wild type | 1 | 20.78 | 0.0000 |
|  | E49P12 | vs | wild type | 1 | 20.78 | 0.0000 |
|  | E49P2 | vs | wild type | 1 | 20.78 | 0.0000 |
|  | E42P4 | vs | wild type | 1 | 20.78 | 0.0000 |
|  | D24P2 | vs | wild type | 1 | 20.78 | 0.0000 |
|  | D39P12 | vs | wild type | 1 | 20.78 | 0.0000 |
|  | E21P2 | vs | wild type | 1 | 20.78 | 0.0000 |
|  | B64P9 | vs | wild type | 1 | 20.78 | 0.0000 |
|  | E23P5 | vs | wild type | 1 | 20.78 | 0.0000 |
|  | E41P10 | vs | wild type | 1 | 20.78 | 0.0000 |
|  | C44P11 | vs | wild type | 1 | 20.78 | 0.0000 |
|  | E33P2 | vs | wild type | 1 | 20.78 | 0.0000 |
|  | E51P12 | vs | wild type | 1 | 20.78 | 0.0000 |
|  | E28P15 | vs | wild type | 1 | 20.78 | 0.0000 |
|  | E35P11 | vs | wild type | 1 | 18.16 | 0.0001 |
|  | C31P12 | vs | wild type | 1 | 18.16 | 0.0001 |
|  | E60P6 | vs | wild type | 1 | 17.68 | 0.0001 |
|  | E47P1 | vs | wild type | 1 | 17.68 | 0.0001 |
|  | D50P5 | vs | wild type | 1 | 17.68 | 0.0001 |
|  | D26P3 | vs | wild type | 1 | 17.68 | 0.0001 |
|  | A47P16 | vs | wild type | 1 | 17.68 | 0.0001 |
|  | D41P3 | vs | wild type | 1 | 17.68 | 0.0001 |
|  | E23P11 | vs | wild type | 1 | 17.68 | 0.0001 |
|  | E21P5 | vs | wild type | 1 | 17 | 0.0001 |
|  | D43P2 | vs | wild type | 1 | 17 | 0.0001 |
|  | E28P5 | vs | wild type | 1 | 17 | 0.0001 |
|  | E31P8 | vs | wild type | 1 | 17 | 0.0001 |
|  | E41P7 | vs | wild type | 1 | 17 | 0.0001 |
|  | E24P17 | vs | wild type | 1 | 17 | 0.0001 |
|  | E21P1 | vs | wild type | 1 | 17 | 0.0001 |

Appendix 21 cont’d

|  | E29P12 | vs | wild type | 1 | 17 | 0.0001 |
| --- | --- | --- | --- | --- | --- | --- |
|  | E33P6 | vs | wild type | 1 | 17 | 0.0001 |
|  | D22P8 | vs | wild type | 1 | 15.94 | 0.0001 |
|  | B47P3 | vs | wild type | 1 | 15.94 | 0.0001 |
|  | E35P2 | vs | wild type | 1 | 15.94 | 0.0001 |
|  | C57P1 | vs | wild type | 1 | 15.15 | 0.0002 |
|  | A48P6 | vs | wild type | 1 | 15.15 | 0.0002 |
|  | E23P12 | vs | wild type | 1 | 15.15 | 0.0002 |
|  | E40P7 | vs | wild type | 1 | 15.15 | 0.0002 |
|  | E30P2 | vs | wild type | 1 | 15.15 | 0.0002 |
|  | C47P13 | vs | wild type | 1 | 15.15 | 0.0002 |
|  | E36P13 | vs | wild type | 1 | 15.15 | 0.0002 |
|  | E27P10 | vs | wild type | 1 | 15.15 | 0.0002 |
|  | C25P7 | vs | wild type | 1 | 15.15 | 0.0002 |
|  | E28P14 | vs | wild type | 1 | 15.15 | 0.0002 |
|  | E48P6 | vs | wild type | 1 | 14.06 | 0.0003 |
|  | B51P18 | vs | wild type | 1 | 14.06 | 0.0003 |
|  | E42P1 | vs | wild type | 1 | 14.06 | 0.0003 |
|  | B51P14 | vs | wild type | 1 | 14.06 | 0.0003 |
|  | C51P4 | vs | wild type | 1 | 14.06 | 0.0003 |
|  | C40P7 | vs | wild type | 1 | 14.06 | 0.0003 |
|  | E37P10 | vs | wild type | 1 | 14.06 | 0.0003 |
|  | E29P11 | vs | wild type | 1 | 14.06 | 0.0003 |
|  | D43P4 | vs | wild type | 1 | 14.06 | 0.0003 |
|  | E24P16 | vs | wild type | 1 | 14.06 | 0.0003 |
|  | E45P7 | vs | wild type | 1 | 14.06 | 0.0003 |
|  | E47P2 | vs | wild type | 1 | 14.06 | 0.0003 |
|  | E30P14 | vs | wild type | 1 | 14.06 | 0.0003 |
|  | D26P6 | vs | wild type | 1 | 14.06 | 0.0003 |
|  | D31P9 | vs | wild type | 1 | 14.06 | 0.0003 |
|  | E25P11 | vs | wild type | 1 | 14.06 | 0.0003 |
|  | E43P5 | vs | wild type | 1 | 14.06 | 0.0003 |
|  | A63P11 | vs | wild type | 1 | 13.07 | 0.0005 |
|  | B51P4 | vs | wild type | 1 | 13.07 | 0.0005 |
|  | B62P6 | vs | wild type | 1 | 13.07 | 0.0005 |
|  | A47P1 | vs | wild type | 1 | 13.07 | 0.0005 |
|  | E36P8 | vs | wild type | 1 | 13.07 | 0.0005 |
|  | C50P2 | vs | wild type | 1 | 12.45 | 0.0007 |
|  | C56P3 | vs | wild type | 1 | 12.45 | 0.0007 |
|  | D21P1 | vs | wild type | 1 | 12.45 | 0.0007 |
|  | E43P8 | vs | wild type | 1 | 12.45 | 0.0007 |

Appendix 21 cont’d

|  | C29P8 | vs | wild type | 1 | 12.45 | 0.0007 |
| --- | --- | --- | --- | --- | --- | --- |
|  | D50P10 | vs | wild type | 1 | 11.74 | 0.0010 |
|  | E40P13 | vs | wild type | 1 | 11.74 | 0.0010 |
|  | E43P10 | vs | wild type | 1 | 11.74 | 0.0010 |
|  | E58P6 | vs | wild type | 1 | 11.74 | 0.0010 |
|  | B61P5 | vs | wild type | 1 | 11.74 | 0.0010 |
|  | D61P3 | vs | wild type | 1 | 11.74 | 0.0010 |
|  | D21P9 | vs | wild type | 1 | 11.74 | 0.0010 |
|  | C3P5 | vs | wild type | 1 | 11.74 | 0.0010 |
|  | E41P2 | vs | wild type | 1 | 11.74 | 0.0010 |
|  | B59P8 | vs | wild type | 1 | 11.74 | 0.0010 |
|  | E25P1 | vs | wild type | 1 | 11.74 | 0.0010 |
|  | E32P1 | vs | wild type | 1 | 11.74 | 0.0010 |
|  | E25P6 | vs | wild type | 1 | 11.74 | 0.0010 |
|  | E46P13 | vs | wild type | 1 | 11.74 | 0.0010 |
|  | C21P3 | vs | wild type | 1 | 11.74 | 0.0010 |
|  | C9P1 | vs | wild type | 1 | 11.74 | 0.0010 |
|  | E41P13 | vs | wild type | 1 | 11.74 | 0.0010 |
|  | E41P1 | vs | wild type | 1 | 11.74 | 0.0010 |
|  | D57P2 | vs | wild type | 1 | 11.74 | 0.0010 |
|  | E36P15 | vs | wild type | 1 | 11.74 | 0.0010 |
|  | D34P10 | vs | wild type | 1 | 11.74 | 0.0010 |
|  | E60P2 | vs | wild type | 1 | 11.34 | 0.0012 |
|  | E30P3 | vs | wild type | 1 | 11.34 | 0.0012 |
|  | C27P2 | vs | wild type | 1 | 11.34 | 0.0012 |
|  | D29P9 | vs | wild type | 1 | 9.88 | 0.0024 |
|  | E50P2 | vs | wild type | 1 | 9.88 | 0.0024 |
|  | B64P8 | vs | wild type | 1 | 9.88 | 0.0024 |
|  | C50P10 | vs | wild type | 1 | 9.88 | 0.0024 |
|  | C59P9 | vs | wild type | 1 | 9.88 | 0.0024 |
|  | E49P1 | vs | wild type | 1 | 9.88 | 0.0024 |
|  | C10P10 | vs | wild type | 1 | 9.88 | 0.0024 |
|  | D41P2 | vs | wild type | 1 | 9.88 | 0.0024 |
|  | B45P13 | vs | wild type | 1 | 9.88 | 0.0024 |
|  | C64P10 | vs | wild type | 1 | 9.88 | 0.0024 |
|  | D24P19 | vs | wild type | 1 | 9.88 | 0.0024 |
|  | E22P4 | vs | wild type | 1 | 9.88 | 0.0024 |
|  | E24P11 | vs | wild type | 1 | 9.88 | 0.0024 |
|  | C37P3 | vs | wild type | 1 | 9.88 | 0.0024 |
|  | C18P10 | vs | wild type | 1 | 9.88 | 0.0024 |
|  | C48P1 | vs | wild type | 1 | 9.88 | 0.0024 |
|  | E47P11 | vs | wild type | 1 | 9.88 | 0.0024 |
|  | E34P11 | vs | wild type | 1 | 9.88 | 0.0024 |
|  | A47P3 | vs | wild type | 1 | 9.88 | 0.0024 |
|  | C26P1 | vs | wild type | 1 | 9.88 | 0.0024 |

Appendix 21 cont’d

|  | E26P11 | vs | wild type | 1 | 9.88 | 0.0024 |
| --- | --- | --- | --- | --- | --- | --- |
|  | E30P16 | vs | wild type | 1 | 9.88 | 0.0024 |
|  | C20P9 | vs | wild type | 1 | 9.88 | 0.0024 |
|  | E48P4 | vs | wild type | 1 | 9.88 | 0.0024 |
|  | E43P11 | vs | wild type | 1 | 9.88 | 0.0024 |
|  | E45P11 | vs | wild type | 1 | 9.88 | 0.0024 |
|  | D26P7 | vs | wild type | 1 | 9.88 | 0.0024 |
|  | E36P16 | vs | wild type | 1 | 9.88 | 0.0024 |
|  | D60P1 | vs | wild type | 1 | 8.37 | 0.0049 |
|  | D35P5 | vs | wild type | 1 | 8.37 | 0.0049 |
|  | D57P15 | vs | wild type | 1 | 8.37 | 0.0049 |
|  | E59P6 | vs | wild type | 1 | 8.37 | 0.0049 |
|  | C63P9 | vs | wild type | 1 | 8.37 | 0.0049 |
|  | D31P8 | vs | wild type | 1 | 8.37 | 0.0049 |
|  | E31P15 | vs | wild type | 1 | 8.37 | 0.0049 |
|  | A47P14 | vs | wild type | 1 | 8.37 | 0.0049 |
|  | A47P17 | vs | wild type | 1 | 8.37 | 0.0049 |
|  | A49P7 | vs | wild type | 1 | 8.37 | 0.0049 |
|  | C40P1 | vs | wild type | 1 | 8.37 | 0.0049 |
|  | D20P7 | vs | wild type | 1 | 8.37 | 0.0049 |
|  | D31P11 | vs | wild type | 1 | 8.37 | 0.0049 |
|  | C29P3 | vs | wild type | 1 | 8.37 | 0.0049 |
|  | C40P12 | vs | wild type | 1 | 8.37 | 0.0049 |
|  | A48P17 | vs | wild type | 1 | 8.37 | 0.0049 |
|  | C37P13 | vs | wild type | 1 | 8.37 | 0.0049 |
|  | D33P10 | vs | wild type | 1 | 8.37 | 0.0049 |
|  | C40P11 | vs | wild type | 1 | 8.37 | 0.0049 |
|  | C41P11 | vs | wild type | 1 | 8.37 | 0.0049 |
|  | E27P8 | vs | wild type | 1 | 8.37 | 0.0049 |
|  | E53P16 | vs | wild type | 1 | 7.94 | 0.0061 |
|  | E40P6 | vs | wild type | 1 | 7.94 | 0.0061 |
|  | D23P1 | vs | wild type | 1 | 7.94 | 0.0061 |
|  | B62P5 | vs | wild type | 1 | 7.94 | 0.0061 |
|  | E24P18 | vs | wild type | 1 | 7.94 | 0.0061 |
|  | E45P5 | vs | wild type | 1 | 7.94 | 0.0061 |
|  | E30P17 | vs | wild type | 1 | 7.94 | 0.0061 |
|  | E47P4 | vs | wild type | 1 | 7.94 | 0.0061 |
|  | E36P3 | vs | wild type | 1 | 7.94 | 0.0061 |
|  | D25P9 | vs | wild type | 1 | 7.94 | 0.0061 |
|  | C29P13 | vs | wild type | 1 | 7.94 | 0.0061 |
|  | C15P10 | vs | wild type | 1 | 7.13 | 0.0092 |

Appendix 21 cont’d

|  | C26P11 | vs | wild type | 1 | 7.13 | 0.0092 |
| --- | --- | --- | --- | --- | --- | --- |
|  | D23P13 | vs | wild type | 1 | 7.13 | 0.0092 |
|  | C25P13 | vs | wild type | 1 | 7.13 | 0.0092 |
|  | D43P13 | vs | wild type | 1 | 7.13 | 0.0092 |
|  | D51P1 | vs | wild type | 1 | 7.13 | 0.0092 |
|  | A47P8 | vs | wild type | 1 | 7.13 | 0.0092 |
|  | C58P13 | vs | wild type | 1 | 7.13 | 0.0092 |
|  | A51P3 | vs | wild type | 1 | 7.13 | 0.0092 |
|  | E41P5 | vs | wild type | 1 | 7.13 | 0.0092 |
|  | E37P2 | vs | wild type | 1 | 7.13 | 0.0092 |
| NOLP | C36P4 | vs | wild type | 1 | 10.37 | 0.0019 |
|  | C36P14 | vs | wild type | 1 | 10.37 | 0.0019 |
|  | C33P8 | vs | wild type | 1 | 10.37 | 0.0019 |
|  | D29P9 | vs | wild type | 1 | 10.37 | 0.0019 |
|  | E50P2 | vs | wild type | 1 | 10.37 | 0.0019 |
|  | A64P7 | vs | wild type | 1 | 10.37 | 0.0019 |
|  | D33P8 | vs | wild type | 1 | 10.37 | 0.0019 |
| NOSP | B64P5 | vs | wild type | 1 | 9.12 | 0.0034 |
|  | C22P13 | vs | wild type | 1 | 9.12 | 0.0034 |
|  | C58P12 | vs | wild type | 1 | 9.12 | 0.0034 |
|  | C60P7 | vs | wild type | 1 | 9.12 | 0.0034 |
|  | D39P12 | vs | wild type | 1 | 9.12 | 0.0034 |
|  | E53P14 | vs | wild type | 1 | 9.12 | 0.0034 |
|  | C50P2 | vs | wild type | 1 | 9.12 | 0.0034 |
|  | C56P3 | vs | wild type | 1 | 9.12 | 0.0034 |
|  | C60P6 | vs | wild type | 1 | 9.12 | 0.0034 |
|  | D1P6 | vs | wild type | 1 | 9.12 | 0.0034 |
|  | D61P1 | vs | wild type | 1 | 9.12 | 0.0034 |
|  | C48P2 | vs | wild type | 1 | 9.12 | 0.0034 |
|  | E21P2 | vs | wild type | 1 | 9.12 | 0.0034 |
|  | B54P7 | vs | wild type | 1 | 9.12 | 0.0034 |
|  | B64P9 | vs | wild type | 1 | 9.12 | 0.0034 |
|  | D22P12 | vs | wild type | 1 | 9.12 | 0.0034 |
|  | E59P7 | vs | wild type | 1 | 9.12 | 0.0034 |
|  | D30P8 | vs | wild type | 1 | 9.12 | 0.0034 |
|  | D61P5 | vs | wild type | 1 | 9.12 | 0.0034 |
|  | E33P11 | vs | wild type | 1 | 9.12 | 0.0034 |
|  | E55P4 | vs | wild type | 1 | 9.12 | 0.0034 |
|  | D22P10 | vs | wild type | 1 | 9.12 | 0.0034 |

Appendix 21 cont’d

|  | B54P5 | vs | wild type | 1 | 9.12 | 0.0034 |
| --- | --- | --- | --- | --- | --- | --- |
|  | E43P15 | vs | wild type | 1 | 9.12 | 0.0034 |
|  | E23P5 | vs | wild type | 1 | 9.12 | 0.0034 |
|  | E41P10 | vs | wild type | 1 | 9.12 | 0.0034 |
|  | E56P9 | vs | wild type | 1 | 9.12 | 0.0034 |
|  | E36P9 | vs | wild type | 1 | 9.12 | 0.0034 |
|  | E51P13 | vs | wild type | 1 | 9.12 | 0.0034 |
|  | C44P11 | vs | wild type | 1 | 9.12 | 0.0034 |
|  | D21P1 | vs | wild type | 1 | 9.12 | 0.0034 |
|  | D25P5 | vs | wild type | 1 | 9.12 | 0.0034 |
|  | E49P5 | vs | wild type | 1 | 9.12 | 0.0034 |
|  | E43P8 | vs | wild type | 1 | 9.12 | 0.0034 |
|  | E36P12 | vs | wild type | 1 | 9.12 | 0.0034 |
|  | E52P12 | vs | wild type | 1 | 9.12 | 0.0034 |
|  | C29P8 | vs | wild type | 1 | 9.12 | 0.0034 |
|  | E33P2 | vs | wild type | 1 | 9.12 | 0.0034 |
|  | A59P7 | vs | wild type | 1 | 12.56 | 0.0007 |
|  | C58P13 | vs | wild type | 1 | 12.56 | 0.0007 |
|  | D28P7 | vs | wild type | 1 | 12.56 | 0.0007 |
|  | D26P5 | vs | wild type | 1 | 12.56 | 0.0007 |
|  | D50P14 | vs | wild type | 1 | 12.56 | 0.0007 |
|  | E53P12 | vs | wild type | 1 | 12.56 | 0.0007 |
|  | E42P8 | vs | wild type | 1 | 12.56 | 0.0007 |
|  | E57P16 | vs | wild type | 1 | 12.56 | 0.0007 |
|  | E58P7 | vs | wild type | 1 | 12.56 | 0.0007 |
|  | C64P3 | vs | wild type | 1 | 12.56 | 0.0007 |
|  | D22P8 | vs | wild type | 1 | 12.56 | 0.0007 |
|  | A51P3 | vs | wild type | 1 | 12.56 | 0.0007 |
|  | B47P3 | vs | wild type | 1 | 12.56 | 0.0007 |
|  | E24P15 | vs | wild type | 1 | 12.56 | 0.0007 |
|  | C20P5 | vs | wild type | 1 | 12.56 | 0.0007 |
|  | E41P5 | vs | wild type | 1 | 12.56 | 0.0007 |
|  | E31P4 | vs | wild type | 1 | 12.56 | 0.0007 |

Appendix 21 cont’d

|  | E35P2 | vs | wild type | 1 | 12.56 | 0.0007 |
| --- | --- | --- | --- | --- | --- | --- |
|  | E37P2 | vs | wild type | 1 | 12.56 | 0.0007 |
|  | E41P8 | vs | wild type | 1 | 12.56 | 0.0007 |
|  | E50P14 | vs | wild type | 1 | 12.56 | 0.0007 |
|  | D44P17 | vs | wild type | 1 | 12.56 | 0.0007 |
|  | E30P12 | vs | wild type | 1 | 12.56 | 0.0007 |
|  | A64P11 | vs | wild type | 1 | 16.56 | 0.0001 |
|  | E51P12 | vs | wild type | 1 | 16.56 | 0.0001 |
|  | E51P16 | vs | wild type | 1 | 16.56 | 0.0001 |
|  | B58P5 | vs | wild type | 1 | 16.56 | 0.0001 |
|  | E45P11 | vs | wild type | 1 | 16.56 | 0.0001 |
|  | E24P12 | vs | wild type | 1 | 16.56 | 0.0001 |
|  | E45P8 | vs | wild type | 1 | 16.56 | 0.0001 |
|  | D28P10 | vs | wild type | 1 | 16.56 | 0.0001 |
|  | E46P1 | vs | wild type | 1 | 16.56 | 0.0001 |
|  | D26P7 | vs | wild type | 1 | 16.56 | 0.0001 |
|  | E28P15 | vs | wild type | 1 | 16.56 | 0.0001 |
|  | E34P16 | vs | wild type | 1 | 16.56 | 0.0001 |
|  | E36P16 | vs | wild type | 1 | 16.56 | 0.0001 |
|  | E43P9 | vs | wild type | 1 | 16.56 | 0.0001 |
|  | E35P9 | vs | wild type | 1 | 16.56 | 0.0001 |
|  | E41P14 | vs | wild type | 1 | 21.11 | 0.0000 |
|  | E51P7 | vs | wild type | 1 | 21.11 | 0.0000 |
|  | D43P12 | vs | wild type | 1 | 21.11 | 0.0000 |
|  | D22P15 | vs | wild type | 1 | 21.11 | 0.0000 |
|  | D22P11 | vs | wild type | 1 | 21.11 | 0.0000 |
|  | E42P13 | vs | wild type | 1 | 21.11 | 0.0000 |
|  | B59P1 | vs | wild type | 1 | 21.11 | 0.0000 |
|  | D44P2 | vs | wild type | 1 | 21.11 | 0.0000 |
|  | D38P7 | vs | wild type | 1 | 21.11 | 0.0000 |
|  | D20P8 | vs | wild type | 1 | 21.11 | 0.0000 |
|  | E49P10 | vs | wild type | 1 | 21.11 | 0.0000 |
|  | C21P11 | vs | wild type | 1 | 26.21 | 0.0000 |
|  | B64P7 | vs | wild type | 1 | 26.21 | 0.0000 |
|  | C54P7 | vs | wild type | 1 | 26.21 | 0.0000 |
|  | D25P10 | vs | wild type | 1 | 26.21 | 0.0000 |
|  | D46P6 | vs | wild type | 1 | 26.21 | 0.0000 |
|  | E23P2 | vs | wild type | 1 | 26.21 | 0.0000 |
|  | C25P12 | vs | wild type | 1 | 26.21 | 0.0000 |
|  | D21P7 | vs | wild type | 1 | 26.21 | 0.0000 |
|  | E49P4 | vs | wild type | 1 | 26.21 | 0.0000 |
|  | E48P10 | vs | wild type | 1 | 26.21 | 0.0000 |
|  | D54P10 | vs | wild type | 1 | 26.21 | 0.0000 |
|  | E49P14 | vs | wild type | 1 | 26.21 | 0.0000 |
|  | D44P3 | vs | wild type | 1 | 26.21 | 0.0000 |
|  | C61P7 | vs | wild type | 1 | 31.86 | 0.0000 |

Appendix 21 cont’d

|  | D62P1 | vs | wild type | 1 | 31.86 | 0.0000 |
| --- | --- | --- | --- | --- | --- | --- |
|  | B63P6 | vs | wild type | 1 | 31.86 | 0.0000 |
|  | D44P18 | vs | wild type | 1 | 31.86 | 0.0000 |
|  | E40P4 | vs | wild type | 1 | 31.86 | 0.0000 |
|  | E43P2 | vs | wild type | 1 | 31.86 | 0.0000 |
|  | C20P13 | vs | wild type | 1 | 38.06 | 0.0000 |
|  | D32P2 | vs | wild type | 1 | 38.06 | 0.0000 |
|  | E57P2 | vs | wild type | 1 | 38.06 | 0.0000 |
|  | E48P9 | vs | wild type | 1 | 38.06 | 0.0000 |
|  | D61P6 | vs | wild type | 1 | 38.06 | 0.0000 |
|  | E43P16 | vs | wild type | 1 | 38.06 | 0.0000 |
|  | A61P9 | vs | wild type | 1 | 44.82 | 0.0000 |
|  | D60P5 | vs | wild type | 1 | 44.82 | 0.0000 |
|  | D60P6 | vs | wild type | 1 | 44.82 | 0.0000 |
|  | E57P8 | vs | wild type | 1 | 44.82 | 0.0000 |
|  | B58P13 | vs | wild type | 1 | 44.82 | 0.0000 |
|  | E57P11 | vs | wild type | 1 | 44.82 | 0.0000 |
|  | E31P5 | vs | wild type | 1 | 52.13 | 0.0000 |
|  | D24P4 | vs | wild type | 1 | 52.13 | 0.0000 |
|  | D32P1 | vs | wild type | 1 | 52.13 | 0.0000 |
|  | E55P11 | vs | wild type | 1 | 52.13 | 0.0000 |
|  | E43P13 | vs | wild type | 1 | 52.13 | 0.0000 |
|  | E57P17 | vs | wild type | 1 | 52.13 | 0.0000 |
|  | E46P10 | vs | wild type | 1 | 52.13 | 0.0000 |
